# The Global Prevalence of HTLV-1 and HTLV-2 Infections among Immigrants and Refugees—A Systematic Review and Meta-Analysis

**DOI:** 10.3390/v16101526

**Published:** 2024-09-27

**Authors:** Thaís Augusto Marinho, Michele Tiemi Okita, Rafael Alves Guimarães, Ana Laura de Sene Amâncio Zara, Karlla Antonieta Amorim Caetano, Sheila Araújo Teles, Márcia Alves Dias de Matos, Megmar Aparecida dos Santos Carneiro, Regina Maria Bringel Martins

**Affiliations:** 1 Institute of Tropical Pathology and Public Health, Federal University of Goiás, Goiânia 74605-050, Goiás, Brazil; thaismarinho@egresso.ufg.br (T.A.M.); okita.micheletiemi@gmail.com (M.T.O.); rafaelalves@ufg.br (R.A.G.); marciaalves@ufg.br (M.A.D.d.M.); megmar@ufg.br (M.A.d.S.C.); 2 Faculty of Nursing, Federal University of Goiás, Goiânia 74605-080, Goiás, Brazil; karlla@ufg.br (K.A.A.C.); sateles@ufg.br (S.A.T.); 3 Health Technology Assessment Center, Federal University of São Paulo, Diadema 09913-030, São Paulo, Brazil; analauraufg@gmail.com

**Keywords:** HTLV-1, HTLV-2, immigrant, refugee, prevalence, systematic review, meta-analysis

## Abstract

This is the first systematic review and meta-analysis to estimate the prevalence of human T-lymphotropic virus 1 and 2 (HTLV-1 and 2) infections among immigrants and refugees worldwide. PubMed/MEDLINE, Scopus, EMBASE, Web of Science, and Virtual Health Library (VHL) databases were searched for studies published from their inception to 6 January 2023. A meta-analysis using a generalized linear mixed model with a random effect was performed for HTLV-1 and HTLV-2. Subgroup analyses were performed based on the decade of study, sample size, confirmatory methods, region of study, risk group, and region of origin. Of the 381 studies initially identified, 21 were included. The pooled prevalence of HTLV-1 and HTLV-2 was 1.28% (95% CI: 0.58, 2.81) and 0.11% (95% CI: 0.04, 0.33), respectively. HTLV-1 prevalence differed significantly by region of origin, with the highest prevalence among those from the Western Pacific Region (7.27%; 95% CI: 2.94, 16.83). The subgroup analysis also showed significant differences between the estimates of HTLV-1 considering the decade of study, sample size, and region of study. For HTLV-2, significant differences were shown in relation to sample size, confirmatory methods, and risk group. The higher HTLV-1 prevalence found deserves public health attention in immigrant and refugee-receiving non-endemic countries.

## 1. Introduction

Human T-lymphotropic virus type 1 (HTLV-1) is a retrovirus that causes adult T-cell leukemia/lymphoma (ATL), HTLV-1-associated myelopathy/tropical spastic paraparesis (HAM/TSP), and other inflammatory diseases [1,2,3]. Additionally, a World Health Organization (WHO) report stresses that HTLV-1 infection is associated with a 57% increase in all-cause mortality [4]. Although some neurological disorders are associated with HTLV-2 [5,6,7], its pathological role is still unclear. HTLV-1 and HTLV-2 are transmitted through unprotected sexual intercourse, transfusion/transplantation of contaminated blood/organs, or injection of drugs and from mother to child, mainly through breastfeeding [3,8,9].

The geographical distribution of HTLV-1 infection indicates that it is endemic in specific regions, including Southern Japan, Northeastern Iran, Romania, sub-Saharan Africa, South America, almost all Caribbean islands, Southeastern regions of the USA, Melanesia, and also in Indigenous Australians [10,11,12,13,14,15,16,17,18,19]. HTLV-2 infection is endemic in American Indigenous populations and people who inject drugs (PWID), as well as in some Indigenous people in the African region [20,21,22,23].

According to the International Organization for Migration (IOM), “migrant” is defined as a broad term that includes various legal categories, including international migrants [24,25]. “Immigrant” is a more specific term that refers to people who may have moved in search of work or an educational opportunity and who intend to settle permanently in their new location [24], whereas refugees are forcibly displaced from their home country and undergo a resettlement process in their new country, receiving protection and permanent residency in the host country [26].

Migratory flows are significant pathways for the spread, emergence, and re-emergence of infectious agents in different geographic areas [27,28,29]. In addition to factors such as the high prevalence of infectious diseases in the country of origin, during transit, and/or at the destination, health systems with limited resources, low vaccination coverage, socioeconomic inequality, high levels of poverty, and ethnic, political, and religious conflicts are related to the vulnerability of international migrants [30,31].

Migratory flows play a significant role in the spread of HTLV-1 and HTLV-2 and have contributed to the origin and spread of these viruses from Africa to Europe, Asia, and the Americas [32,33]. To date, no systematic review has assessed the prevalence of HTLV-1/2 among international migrants. Therefore, this study aimed to estimate the prevalence of HTLV-1 and HTLV-2 infections among immigrants and refugees worldwide.

## 2. Materials and Methods

### 2.1. Study Design and Search Strategy

This systematic review with meta-analysis was conducted according to the “Preferred Reporting Items for Systematic Reviews and Meta-Analyses” (PRISMA) guidelines (Table S1) [34]. The systematic review protocol was registered in the International Prospective Registry of Systematic Reviews (PROSPERO), with registration number CRD42021293373 (https://www.crd.york.ac.uk/prospero/display_record.php?RecordID=293373).

We searched PubMed/MEDLINE, Scopus, EMBASE, Web of Science, and Virtual Health Library (VHL) databases for articles published up to 6 January 2023. The search strategy is detailed in the Supplementary Materials (Table S2). Bibliographic references of included studies were examined to identify additional publications.

The Boolean search strategy used in this study combined PEO-style keywords [Population (P), Exposure (E), and Outcome (O)] [35,36]. The descriptors selected for (P) included the Medical Subject Headings (MeSH) terms and subcategories related to “immigrants” and “refugees”, for (E), the terms “HTLV-1 and HTLV-2 infections” and variations thereof were used, and for (O), the terms “prevalence”, “epidemiology”, and “cross-sectional studies” were used.

### 2.2. Study Selection

Original observational studies were considered for inclusion in the systematic search, which included the prevalence of HTLV-1 and/or HTLV-2 among immigrants and refugees, using screening and confirmatory tests for HTLV-1/2 (immunofluorescence assay/IFA, Western blot/WB or line immunoassay/LIA, and/or polymerase chain reaction/PCR). There were no restrictions on language, year of publication, or geographic region of the studies. Publications were excluded when they were classified as editorials, letters to the editor, systematic and non-systematic literature reviews, meta-analyses, case reports, case series, and clinical trials. Studies published in non-English language journals were translated prior to evaluation. All publications identified in the databases were exported to the Mendeley^®^ reference manager (Mendeley^®^, Elsevier^®^, version 1.19.5/2019) to remove duplicates.

After removing duplicates, all publications were exported to Rayyan^®^ software (Rayyan QCRI, Qatar Computing Research Institute—Data Analytics) [37] for the selection process (Step I) based on titles and abstracts, and the confirmation of eligibility (Step II) through a full-text review. The selection of publications was conducted by two reviewers.

### 2.3. Data Extraction

All studies that met the eligibility criteria were included in this review and critically evaluated. Study data were collected and managed using Research Electronic Data Capture (REDCap) platform tools hosted at the Federal University of Goiás, Brazil [38,39]. The instrument was designed to extract relevant data from the articles and included (i) study identification: first author, journal, and year of publication; (ii) study characteristics: study design, sample size, decade of study, migration category, gender, age, region, country and setting of collection and region of origin; and (iii) laboratory assays and epidemiological data: HTLV-1/2 screening and confirmatory methods and prevalence of HTLV-1 and/or HTLV-2. Data were extracted independently by two reviewers, with any disagreements resolved by consensus after discussion with a third reviewer.

### 2.4. Risk of Bias Assessment

The Joanna Briggs Institute (JBI) Critical Review Checklist for Studies Reporting Prevalence Data was used to assess the risk of bias in the included studies [35]. The risk of biased judgments were independently verified by two reviewers, and disagreements were discussed until consensus was reached with a third reviewer.

### 2.5. Data Synthesis and Analysis

Meta-analysis was conducted using R language, version 4.0.2 [40], using the “meta” [41] and “metafor” [42] packages. Random effects models were used to estimate the prevalence of HTLV-1 and HTLV-2, using a Generalized Linear Mixed Model (GLMM) as a method to group the studies and logit transformation as a summary measurement method [43]. The logistic model with random effects showed good performance for meta-analyses of binomial data for rare events. The 95% confidence interval (95% CI) for the individual studies was calculated using a simple approximation interval with continuity correction. The pooled prevalence of HTLV-1 and HTLV-2 was determined using a random effects model due to the presence of high (HTLV-1) and moderate (HTLV-2) heterogeneity. Heterogeneity between studies was assessed using *I*^2^ statistics, where *I*^2^ values of 60–100%, 40–59%, and 0–39% indicate high, moderate, and low heterogeneity, respectively [44]. Heterogeneity was also assessed using chi-square (χ^2^) statistics, with *p*-values < 0.05 indicating significant heterogeneity.

In addition, we performed subgroup analyses by decade of study, sample size, confirmatory methods, region of study, and risk group for prevalence of HTLV-1 and HTLV-2 infections separately. Subgroups referring to the decade of study were categorized as before 1992, 1992–2001, 2002–2011, and 2012–2021. For sample size, subgroups <640 and ≥640 were defined based on the median number of samples from all studies included in this review. Confirmatory methods included four subgroups (WB/LIA and PCR, WB, PCR or qPCR, and IFA) based on the methods used in their respective studies. To classify the region of study, the world geographic regions established by the WHO were adopted: the African Region, the Region of the Americas, the Eastern Mediterranean Region, the European Region, the South-East Asia Region, and the Western Pacific Region [45]. Analysis was also conducted by risk group according to the study population. For the high- and low-risk subgroups, studies on immigrant and refugee populations at high risk of HTLV-1/2, such as sex workers [46,47], prisoners [48], HIV-positive individuals [49], and Indigenous people [50], were included in the first subgroup, while the other studies were included in the second subgroup. Subgroup analyses for sex and age could not be performed due to the high proportion of missing data for these variables in the studies. For the age of immigrants and refugees, five studies reported only the median age of their participants, and the average age was then estimated [51].

Another independent subgroup analysis was conducted for HTLV-1 and HTLV-2 estimates by region of origin. This was necessary due to the different origins of international migrants in the same study, resulting in different numerators and denominators for this variable. To classify the regions of origin, the world geographic regions established by the WHO were also adopted [45].

## 3. Results

### 3.1. Study Selection and Characteristics

**Figure 1:**
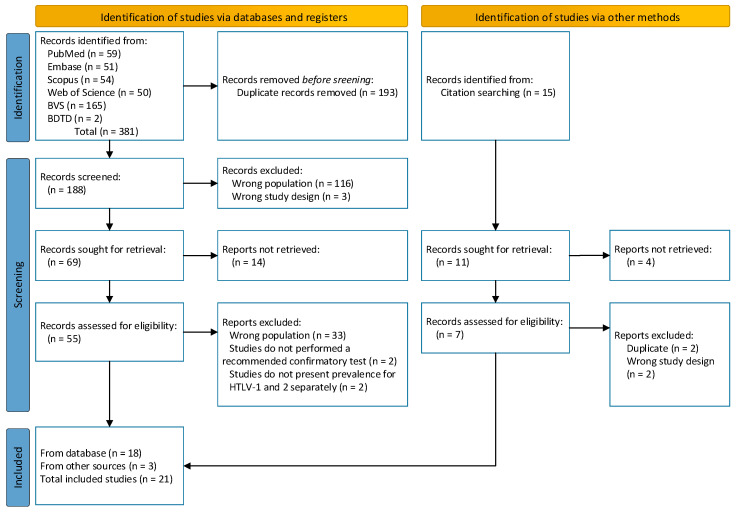
PRISMA flow diagram.

The search strategy resulted in 381 records. After removing duplicates, 193 unique records were screened by title and abstract. A total of 188 full-text articles were evaluated for eligibility, of which 119 were excluded. This resulted in 69 research-eligible articles. Of these, 14 reports were not retrievable. Among 55 records assessed for eligibility, 33 studies were excluded for not addressing the target population. Additionally, two studies were excluded for not performing a recommended confirmatory test and two others were excluded for not presenting the prevalence of HTLV-1 and HTLV-2 separately. Three studies were included through a manual search. In the end, 21 articles were included (Figure 1); thirteen articles present data for HTLV-1 and HTLV-2 and eight present data for HTLV-1 only.

The characteristics of the included studies are presented in Table 1. Regarding the geographic areas where the studies were carried out, nine studies were conducted in the European Region: three in Italy [48,49,52], five in Spain [46,53,54,55,56], and one in England [57]. Only one study was conducted in the Eastern Mediterranean Region, specifically in Israel [58]. In the Region of the Americas, 11 studies were conducted. In North America, four studies were conducted: three in the USA [59,60,61], with one of these in Hawaii [61], and one in Canada [62]. In South America, seven investigations were conducted: four in Brazil [50,63,64,65], one in Argentina [47], one in Peru [66] and one in Bolivia [67].

Of the total, only three studies included refugees: two in the Region of the Americas, one in the USA involving refugees from the South-East Asian Region [59], another in Brazil involving Indigenous Venezuelan refugees [50], and a third in the European Region, involving immigrants and refugees in Italy from African, Eastern Mediterranean, and South-East Asia Regions [52].

Study settings varied and included cities (n = 4, one of which also included a prison), clinics (n = 6, one of which was specifically for health support for refugees), hospitals (n = 4), one of which included both hospitals and a clinic, blood bank (n = 1), home visits and telephone calls (n = 1), sex work locations (n = 2), and Japanese communities (n = 3).

The average age ranged from 26 years among immigrants and refugees in Italy from African, Eastern Mediterranean, and South-East Asia regions [52] to 72.5 years among immigrants in Hawaii from Japan [61]. Fourteen investigated more than 50% of women [46,47,48,49,50,53,54,55,56,59,60,62,65,67]. Three studies included only women: one with female sex workers and two with pregnant women [47,55,56].

Of the 21 articles investigated, chemiluminescent immunoassay (CLIA) was used in the laboratory screening of anti-HTLV-1/2 in two [52,53], particle agglutination (PA) was used in one [67], and enzyme-linked immunosorbent assay (ELISA) was used in 18 [45,46,47,48,49,53,54,55,56,57,58,59,60,61,62,63,64,65], two of which used a combination of ELISA and PA [47,57] and one used ELISA and radioimmunoassay (RIA) combined [60]. Regarding confirmatory methods, WB was used in 10 studies [46,47,53,54,55,59,60,61,66,67], IFA was used in two [57,62], a combination of WB or LIA with PCR was used in seven [48,49,52,56,58,63,64], and PCR or real-time PCR/qPCR was used in two [50,65]. The authors used these tests to detect HTLV-1 infection in all studies, whereas HTLV-2 was detected in 13 studies [46,47,48,49,50,52,53,54,55,56,62,63,64].

### 3.2. Prevalence of HTLV-1 and HTLV-2

**Figure 2:**
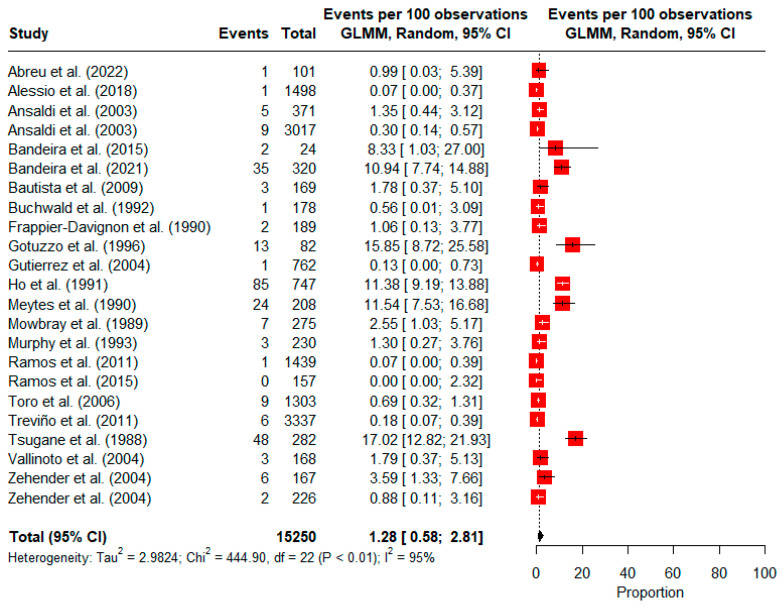
Forest plot of the prevalence of HTLV-1 infection among immigrants and refugees [46,47,48,49,50,52,53,54,55,56,57,58,59,60,61,62,63,64,65,66,67].

A total of 21 studies were included in the meta-analysis for HTLV-1. Two studies included two migrant groups; thus, prevalences were calculated separately for each group: a case–control study [49] presenting estimates stratified by HIV-1-positive and HIV-1-negative immigrants and a cross-sectional study [48] where estimates were stratified by open-population immigrants and immigrant inmates. The overall pooled prevalence of HTLV-1 was 1.28% (95% CI: 0.58, 2.81; 15,250 observations; and 267 events). High heterogeneity was observed among the included studies (*I*^2^ = 95%), with a χ^2^ value of 444.90 (*p* < 0.01). Estimates ranged widely from 0% [53] to 17.02% [67] (Figure 2).

**Figure 3:**
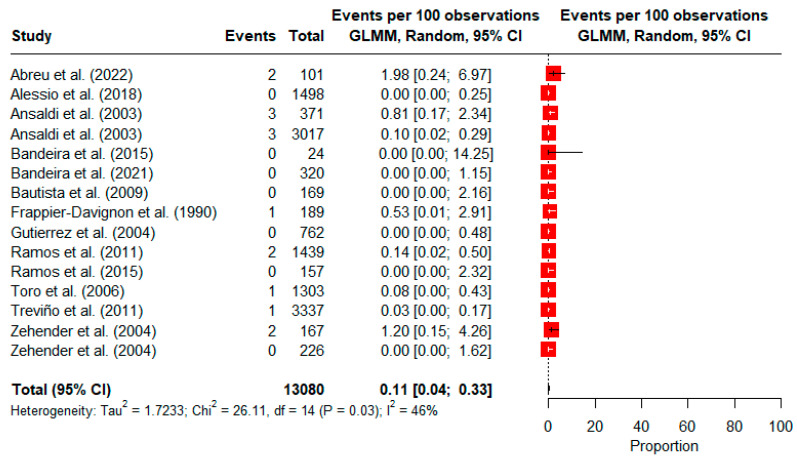
Forest plot of the prevalence of HTLV-2 infection among immigrants and refugees [46,47,48,49,50,52,53,54,55,56,62,63,64].

Regarding HTLV-2, 13 studies were included in the meta-analysis. Similar to HTLV-1, in two studies [48,49], prevalences were obtained separately for each migrant group. The overall pooled prevalence of HTLV-2 was 0.11% (95% CI: 0.04, 0.33; 13,080 observations; and 15 events). Moderate heterogeneity was observed among the included studies (*I*^2^ = 46%), with a χ^2^ of 26.11 (*p* = 0.03). Estimates ranged from 0% in seven studies [46,47,49,52,53,63,64] to 1.98% in Indigenous refugees from Venezuela in Brazil [50] (Figure 3).

### 3.3. Subgroup Analyses

To explain the heterogeneity between studies, subgroup analyses were performed. For HTLV-1, estimates are presented by decade of study (Figure S1), sample size (Figure S2), confirmatory methods (Figure S3), region of study (Figure S4), and risk group (Figure S5). For HTLV-2, estimates are also shown by these subgroups (Figures S6–S10, respectively). As presented in Table 2, significant differences were noted in the estimated prevalence of HTLV-1 among immigrants and refugees considering the decade of study, sample size, and region of study (*p* < 0.01). The subgroup analysis of HTLV-2 prevalence (Table 3) showed a significant difference regarding sample size (*p* < 0.01), confirmatory methods (*p* < 0.01), and risk group (*p* = 0.03).

An independent subgroup analysis of the estimates of HTLV-1 and HTLV-2 was conducted by region of origin of immigrants and refugees (Table 4). The analysis showed that heterogeneity by region of origin was only statistically significant for HTLV-1 (*p* < 0.01), with the highest prevalence among those from the Western Pacific Region.

### 3.4. Assessment of Risk of Bias

There was a high risk of bias among the included studies, with twenty cross-sectional studies and one case–control study. These studies received a score of 5 out of 8 using the Joanna Briggs Institute (JBI) Critical Appraisal Checklist for Analytical Cross Sectional Studies and a score of 5 out of 10 using the JBI Critical Appraisal Checklist for Case Control Studies (Tables S3 and S4, respectively).

## 4. Discussion

This systematic review with meta-analysis is the first to show the pooled prevalence of HTLV-1 and HTLV-2 infections in immigrants and refugees worldwide. These data are important for public health, considering that these retrovirus-associated diseases remain neglected infections in many countries [68,69,70,71]. Additionally, immigrants and refugees constitute diverse, mobile, and vulnerable populations that confront barriers to accessing healthcare systems [27,29,30]. Moreover, most people living with HTLV-1/2 are asymptomatic and unaware of their infection and, thus, these carriers are potential viral disseminators, particularly migrants from high-prevalence countries [72].

It was observed that the overall pooled prevalence of HTLV-1 infection among immigrants and refugees (1.28%; 95% CI: 0.58, 2.81) was higher than that estimated for HTLV-2 (0.11%; 95% CI: 0.04, 0.33). Although it is challenging to conduct a meta-analysis to estimate the global prevalence of these infections among international migrants from various regions and countries worldwide, these pooled estimates should be interpreted with caution considering the heterogeneity observed.

It is notable in the subgroup analysis that estimates of HTLV-1 by decade of study were lower in studies conducted in more recent decades (2012–2021: 0.81%; 2002–2011: 0.23%) in relation to the older ones (before 1992: 4.88%; 1992–2001: 1.17%; *p* < 0.01). This decrease is likely due to improvements in laboratory technology [73], as well as improvements in research designs [74], and the implementation in some countries of policies addressing HTLV-1 infection and its prevention. In this regard, various policies have been implemented in Japan over the years [75,76,77]. Additionally, the prevalence of HTLV-1 was lower in studies with larger sample sizes compared to those with smaller samples (≥640: 0.31% vs. <640: 2.62%; *p* < 0.01). These data are consistent with those reported in a previous meta-analysis, in which studies with large sample sizes tended to report a lower prevalence of HTLV-1 infection than those with small sample sizes [78]. Sample size significantly affects reliability in observational studies. As the sample size increases, the confidence intervals of estimates decrease, and there is a possibility of detecting differences between subgroups [74].

A significant difference in pooled HTLV-1 estimates was also observed among regions of origin, ranging from 0.07% among immigrants and refugees from the European Region to 7.27% in those from the Western Pacific Region. Although six studies did not report the origins of some immigrants, they observed no HTLV-1/2 positive results. Notably, most immigrants from the Western Pacific Region came from HTLV-1-endemic areas in Japan to North and South America in the 20th century [60,61,63,64,65,66,67,79], a detail that aligns with serological and molecular evidence that suggests that migratory flows from Japan have played a role in the introduction of HTLV-1 in some regions of Brazil [32,63,64,65].

According to the study region, HTLV-1 prevalence ranged from 0.52% in the European Region to 11.54% in the Eastern Mediterranean Region, though only one study was conducted there, and in the European Region, studies were limited to Italy [48,49,52], Spain [46,53,54,55,56], and England [57]. In addition, it is important to consider the currently intense waves of migration to Europe [27,29]. In Spain, a non-endemic country experiencing a high migrant flow from Latin America and Sub-Saharan Africa [46,53,54,55,56], HAM/TSP is the most frequent clinical manifestation of HTLV-1 infection (incidence of 2–3 new cases per year), with middle-aged female migrants from Latin America being the most affected [80]. Similarly, the current average incidence of ATL in Spain (2 cases per year) is mostly associated with migrants from Latin America (57%), followed by those from Africa (26%) [81]. Thus, expanding HTLV-1/2 testing to target populations is necessary, including international immigrants and refugees coming from endemic countries, as well as natives who had lived in, have mothers from, or have sexual partners from such countries [80,81].

In this line, integrating HTLV-1 control with other prevention strategies should be considered due to its clinical relevance. In addition, there is no protective vaccine or effective antiviral therapy for this lifelong infection. In 2022, HTLV-1 was included in the WHO’s strategic planning for sexually transmitted infection (STI) control for 2030 [82]. To achieve this goal, it is essential to facilitate early diagnosis of most asymptomatic carriers so that they can be clinically monitored and counseled about transmission-preventive measures [83].

HTLV-2 prevalence studies in this review showed moderate heterogeneity. In addition to sample size, a significant difference in subgroup analysis was noted for confirmatory methods (*p* < 0.01). This was consistent with that reported elsewhere [84], which revealed that the heterogeneity was lower in studies that used only WB for HTLV-2, whereas it was lower in studies using only PCR for HTLV-1, suggesting that the type of confirmatory test used appears to influence results differently for each type of HTLV. Our study validates this assumption. A significant difference in subgroup analysis by risk group was also observed for HTLV-2 (low- and high-risk subgroups: 0.07% vs. 0.40%; *p* = 0.03). In this high-risk subgroup, five studies included sex workers, prisoners, HIV-positive individuals, and Indigenous people. Notably, the highest HTLV-2 prevalence was in Venezuelans of the Warao ethnic group living as refugees in Belém, a major city in the Brazilian Amazon [50]. Of note, this intense migratory flow from Venezuela to other Latin American countries has been motivated by political, social, and economic crises in recent years [85], and it may contribute to the dispersion of HTLV-1/2 [50].

Despite global efforts to assess HTLV-1 and HTLV-2 prevalence among international migrants, significant gaps remain. Notably, only 3 of the 21 studies (14.3%) analyzed reported HTLV-1/2 prevalence in refugees [50,52,59]. Additionally, gaps in representative immigrant samples hinder updates on HTLV-1/2 prevalence in the Eastern Mediterranean Region [58], as well as other WHO regions (African Region, Southeast Asia Region, and Western Pacific Region) given the data scarcity.

This systematic review with meta-analysis presents other limitations regarding the search strategy, as it is plausible that some authors provided HTLV-1 and/or HTLV-2 prevalence data for migrants without using the term immigrants but who could still be categorized as such for the purposes of this review. Therefore, the absence of these studies may have resulted in an incomplete data set. Comparability of estimates could also be noted, as different laboratory tests were used and the specificity and sensitivity of anti-HTLV 1/2 assays have improved over time. Additionally, estimates varied across specific vulnerable migrant groups, such as sex workers, HIV-positive individuals, prisoners, and Indigenous people, contributing to the heterogeneity observed.

Despite these limitations, this review provides relevant epidemiological data on HTLV-1 and HTLV-2 infections in immigrants and refugees worldwide. Nevertheless, further epidemiological studies on HTLV-1/2 are necessary to understand the burden of these infections among international migrants in all WHO regions and to guide the development of public policies on educational and prophylactic measures to increase awareness of HTLV-1 and HTLV-2 infections and reduce viral transmission and infection-related diseases.

In conclusion, the results of this systematic review and meta-analysis show that, despite the heterogeneity observed, the pooled HTLV-1 prevalence among immigrants and refugees is higher than HTLV-2. The high prevalence of HTLV-1 found, particularly among those from the Western Pacific Region, suggests that targeted screening of international migrants from HTLV-1 endemic regions could be a significant public health intervention for HTLV-1 infection control in immigrant and refugee-receiving non-endemic countries.

## Figures and Tables

**Table 1:** Summary of data from selected articles on HTLV-1 and HTLV-2 prevalence among international migrants.

First Author, Year of Publication	Study Design	Region of Study (Country)	Region of Origin (WHO)	Migratory Status	Setting	Year of Study	Screening Method	Confirmatory Method	Sample Size (N)	F (%)	M (%)	Mean Age (Years)	HTLV1 (%)	HTLV2 (%)
		European Region												
Alessio et al., 2018 [52]	Cross-sectional	Italy	African Region, Eastern Mediterranean Region, South-East Asia Region	Immigrants and refugees	City	2012–2017	CLIA	WB, PCR	1498	10.9	89.0	26	0.07	0
Ramos et al., 2015 [53]	Cross-sectional	Spain	Region of the Americas	Immigrants	Hospital	2012–2014	CLIA	WB	180 157 *	68.3	31.7	38	0	0
Ramos et al., 2011 [55]	Cross-sectional	Spain	European Region, Region of the Americas, African Region, and others not reported/unclear	Immigrant pregnant women	Hospital	2006–2009	ELISA	WB	1439	100	0	30.7	0.07	0.14
Treviño et al., 2011 [56]	Cross-sectional	Spain	South-East Asia Region, European Region, African Region, Region of the Americas, and others not reported/unclear	Immigrant pregnant women	Clinics	2009–2010	ELISA	WB; PCR to indeterminate WB	3337	100	0	29	0.17	0.02
Toro et al., 2006 [54]	Cross-sectional	Spain	European Region, African Region, Region of the Americas, and others not reported/unclear	Immigrants	Hospitals and Clinic	2002–2003	ELISA	WB	1303	62	38	29.5	0.7	0.1
Gutierrez et al., 2004 [46]	Cross-sectional	Spain	European Region, Region of the Americas, African Region	Immigrant sex workers	Working location	1998–2003	ELISA	WB	762	91.7	8.3	27	0.2	0
Zehender et al., 2004 [49]	Case-control	Italy	African Region, Region of the Americas, and others not reported/unclear	HIV-1 positive immigrants	Clinic	1996–2003	ELISA	WB, PCR	167	34.7	65.3	34.3	3.6	1.2
		Italy	African Region, Region of the Americas, and others not reported/unclear	HIV-1 negative immigrant women	Clinic	1996–2003	ELISA	WB, PCR	226	100	0	28.3	0.9	0
Ansaldi et al., 2003 [48]	Cross-sectional	Italy	Region of the Americas, European Region, African Region, and others not reported/unclear	Open-population immigrants	City	1996–2000	ELISA	LIA, WB, PCR	3017	56.6	43.4	NA	0.3	0.1
		Italy	Region of the Americas, European Region, African Region, and others not reported/unclear	Immigrant inmates	Prison	1996–2000	ELISA	LIA, WB, PCR	371	30.7	69.3	NA	1.4	0.8
Mowbray et al., 1989 [57]	Cross-sectional	England	Region of the Americas, South-East Asia Region, and others not reported/unclear	Immigrants	Clinics	NA	PA, ELISA	IFA	275	28.7	71.3	40	2.5	NA
		Eastern Mediterranean Region												
Meytes et al., 1990 [58]	Cross-sectional	Israel	Eastern Mediterranean Region	Immigrants	Blood bank	1988–1989	ELISA	WB, PCR	208	NA	NA	NA	11.5	NA
		Region of the Americas (North America)												
Murphy et al., 1993 [60]	Cross-sectional	USA	Western Pacific Region	Immigrants	Clinic and laboratory	1990	ELISA	WB	230	67.4	32.6	NA	1.3	NA
Buchwald et al., 1992 [59]	Cross-sectional	USA	South-East Asia Region	Refugees	Refugee clinic	NA	ELISA	WB	193 178 *	52	48	42	0.6	NA
Ho et al., 1991 [61]	Cross-sectional	USA	Western Pacific Region	Immigrants	Hospital	1967–1975	ELISA, RIA	WB	747	0	100	72.5	11.4	NA
Frappier-Davignon et al.,1990 [62]	Cross-sectional	Canada	Region of the Americas	Immigrants	Home visits/telephone calls	1982	ELISA	IFA	189	57.1	42.9	34.8 (F) 37.6 (M)	1.0	0.5
		Region of the Americas (South America)												
Abreu et al. 2022 [50]	Cross-sectional	Brazil	Region of the Americas	Indigenous refugees	City	2020–2021	ELISA	qPCR	101	57.4	42.6	36	1.0	2.0
Bandeira et al., 2021 [63]	Cross-sectional	Brazil	Western Pacific Region	Immigrants	Japanese communities	2017	ELISA	PCR, WB	320	NA	NA	NA	10.9	0
Bandeira et al., 2015 [64]	Cross-sectional	Brazil	Western Pacific Region	Immigrants	Japanese community	2012–2013	ELISA	WB, PCR	24	NA	NA	NA	8.3	0
Bautista et al. 2009 [47]	Cross-sectional	Argentina	Region of the Americas	Immigrant female sex workers	Working locations	2000–2002	ELISA, PA	WB	169	100	0	NA	1.8	0
Vallinoto et al., 2004 [65]	Cross-sectional	Brazil	Western Pacific Region	Immigrants	City	1999	ELISA	PCR	168	61.9	38.1	NA	1.8	NA
Gotuzzo et al., 1996 [66]	Cross-sectional	Peru	Western Pacific Region	Immigrants	Clinic	1993–1994	ELISA	WB	82	NA	NA	NA	15.8	NA
Tsugane et al., 1988 [67]	Cross-sectional	Bolivia	Western Pacific Region	Immigrants	Japanese communities	1986	PA	WB	282	50.4	49.6	54.7	17.0	NA

F, female; M, male; NA, not available; CLIA, chemiluminescent immunoassay; ELISA, enzyme-linked immunosorbent assay; IFA, immunofluorescence assay; LIA, line immunoassay; PA, particle agglutination; PCR, polymerase chain reaction; RIA, radioimmunoassay; qPCR, real-time PCR; WB, western blot. * Number of samples tested.

**Table 2:** Subgroup analysis assessing the pooled prevalence of HTLV-1 and sources of heterogeneity.

Subgroups	Categories	N°. of Studies	Sample Size	Prevalence		*I*^2^ (%)	*X*^2^ (*p*-Value between Subgroups)
				%	95% CI		
Decade of study	Before 1992	6	1931	4.88	1.44, 15.30	91	18.48 **(<0.01)**
	1992–2001	9	5140	1.17	0.38, 3.49	92	
	2002–2011	3	6079	0.23	0.02, 2.23	79	
	2012–2021	5	2100	0.81	0.03, 16.37	87	
Sample size	<640	16	3147	2.62	1.27, 5.31	88	7.40 **(<0.01)**
	≥640	7	12,103	0.31	0.05, 1.76	98	
Confirmatory methods	WB	10	5349	1.15	0.24, 5.39	95	0.69 (0.88)
	WB/LIA and PCR	9	9168	1.32	0.32, 5.25	96	
	PCR or qPCR	2	269	1.49	0.00, 90.09	0	
	IFA	2	464	1.94	0.03, 58.76	18	
Region of study	Eastern Mediterranean Region	1	208	11.54	7.53, 16.68	*	48.35 **(<0.01)**
	European Region	13	12,806	0.52	0.21, 1.27	84	
	Region of Americas	9	2236	3.83	1.32, 10.60	87	
Risk group	High	5	1570	1.05	0.27, 3.99	62	0.16 (0.69)
	Low	18	13,680	1.37	0.51, 3.59	96	

Bold-value, *p* < 0.05; CI, confidence interval; *I*^2^, heterogeneity; *X*^2^, chi-square; WB, western blot; LIA, line immunoassay; PCR, polymerase chain reaction; qPCR, real-time PCR; IFA, immunofluorescence assay. * Heterogeneity not generated due to the presence of only one study.

**Table 3:** Subgroup analysis assessing the pooled prevalence of HTLV-2 and sources of heterogeneity.

Subgroups	Categories	N°. of Studies	Sample Size	Prevalence		*I*^2^ (%)	*X*^2^ (*p*-Value between Subgroups)
				%	95% CI		
Decade of study	Before 1992	1	189	0.53	0.01, 2.91	*	4.20 (0.24)
	1992–2001	6	4712	0.16	0.03, 1.04	49	
	2002–2011	3	6079	0.07	0.01, 0.56	0	
	2012–2021	5	2100	0.01	0.00, 62.93	0	
Sample size	<640	9	1724	0.40	0.11, 1.39	0	7.99 **(<0.01)**
	≥640	6	11,356	0.06	0.02, 0.16	0	
Confirmatory methods	WB	5	3830	0.08	0.02, 0.39	0	14.90 **(<0.01)**
	WB/LIA and PCR	8	8960	0.09	0.01, 0.57	56	
	PCR or qPCR	1	101	1.98	0.24, 6.97	*	
	IFA	1	189	0.53	0.01, 2.91	*	
Region of study	European Region	11	12,301	0.09	0.03, 0.30	45	1.17 (0.28)
	Region of Americas	4	779	0.29	0.01, 6.39	0	
Risk group	High	5	1570	0.40	0.05, 2.89	0	4.74 **(0.03)**
	Low	10	11,510	0.07	0.03, 0.15	0	

Bold-value, *p* < 0.05; CI, confidence interval; *I*^2^, heterogeneity; *X*^2^, chi-square; WB, western blot; LIA, line immunoassay; PCR, polymerase chain reaction; qPCR, real-time; IFA, immunofluorescence assay. * Heterogeneity not generated due to the presence of only one study.

**Table 4:** Subgroup analysis assessing the pooled prevalence of HTLV-1 and HTLV-2 by region of origin of immigrants and refugees as a source of heterogeneity.

Region of Origin	No. of Studies	Sample Size	Prevalence		*I*^2^ (%)	*X*^2^ (*p*-Value between Subgroups)
			%	95% CI		
HTLV-1						
African Region	9	5246	0.25	0.07, 0.89	67	39.39 **(<0.01)**
Eastern Mediterranean Region	2	351	0.94	0.00, 100.00	0	
European Region	6	1632	0.07	0.01, 5.15	0	
Region of Americas	13	5021	0.76	0.33, 1.70	75	
South-East Asia Region	4	568	0.18	0.01, 4.09	0	
Western Pacific Region	7	1853	7.27	2.94, 16.83	84	
Not Reported/Unclear	6	579	0.00	0.00, 100.00	0	
HTLV-2						
African Region	9	5246	0.08	0.02, 0.24	0	0.34 (1.00)
Eastern Mediterranean Region	1	143	0.00	0.00, 100.00	*	
European Region	6	1632	0.12	0.01, 2.14	0	
Region of Americas	12	4832	0.12	0.02, 0.69	0	
South-East Asia Region	2	319	0.00	0.00, 100.00	0	
Western Pacific Region	2	344	0.0	0.00, 100.00	0	
Not Reported/Unclear	5	564	0.00	0.00, 100.00	0	

Bold-value, *p* < 0.05; CI, confidence interval; *I*^2^, heterogeneity; *X*^2^, chi-square. * Heterogeneity not generated due to the presence of only one study.

## Supplementary Materials

The following supporting information can be downloaded at: https://www.mdpi.com/article/10.3390/v16101526/s1. Table S1: Preferred Reporting Items For Systematic Reviews and Meta-analyses (PRISMA) 2020 checklist; Table S2: Search strategies from the PubMed, Scopus, EMBASE, Web of Science, and VHL databases; Table S3: Assessment of quality of HTLV-1/2 prevalence studies in international migrants included in this systematic review according to the JBI Critical Appraisal Checklist for Analytical Cross Sectional Studies; Table S4: Assessment of quality of studies of HTLV-1/2 prevalence in immigrants included in the systematic review according to the JBI Critical Appraisal Checklist for Case Control Studies. Figure S1: Forest plot of HTLV-1 prevalence in immigrants and refugees by decade of study. Figure S2: Forest plot of HTLV-1 prevalence in immigrants and refugees by sample size. Figure S3: Forest plot of HTLV-1 prevalence in immigrants and refugees by confirmatory method used. Figure S4: Forest plot of HTLV-1 prevalence in immigrants and refugees by region of study. Figure S5: Forest plot of HTLV-1 prevalence in immigrants and refugees by low- and high-risk groups. Figure S6: Forest plot of HTLV-2 prevalence in immigrants and refugees by decade of study. Figure S7: Forest plot of HTLV-2 prevalence in immigrants and refugees by sample size. Figure S8: Forest plot of HTLV-2 prevalence in immigrants and refugees by confirmatory method used. Figure S9: Forest plot of HTLV-2 prevalence in immigrants and refugees by region of study. Figure S10: Forest plot of HTLV-2 prevalence in immigrants and refugees by low- and high-risk groups.
