# Supplementary material for: The Global Prevalence of HTLV-1 and HTLV-2 Infections among Immigrants and Refugees—A Systematic Review and Meta-Analysis
Source: Viruses. 2024 Sep 27;16(10):1526. doi: 10.3390/v16101526 (PMC11512286; doi:10.3390/v16101526)

Figure S1. Forest plot of HTLV-1 prevalence in immigrants and refugees by decade of study.

Study or Subgroup	Events	Total	Events per 100 observations GLMM, Random, 95% CI
Decade of study = Before 1992			
Tsugane et al. (1988)	48	282	17.02 [12.82; 21.93]
Mowbray et al. (1989)	7	275	2.55 [1.03; 5.17]
Frappier-Davignon et al. (1990)	2	189	1.06 [0.13; 3.77]
Meytes et al. (1990)	24	208	11.54 [7.53; 16.68]
Ho et al. (1991)	85	747	11.38 [9.19; 13.88]
Murphy et al. (1993)	3	230	1.30 [0.27; 3.76]
Total (95% CI)		1931	4.88 [1.44; 15.30]
Heterogeneity: $Tau^2 = 1.2748$; $Chi^2 = 53.49$, df = 5 (P < 0.01); $I^2 = 91\%$			
Decade of study = 1992-2001			
Buchwald et al. (1992)	1	178	0.56 [0.01; 3.09]
Gotuzzo et al. (1996)	13	82	15.85 [8.72; 25.58]
Ansaldi et al. (2003)	5	371	1.35 [0.44; 3.12]
Ansaldi et al. (2003)	9	3017	0.30 [0.14; 0.57]
Vallinoto et al. (2004)	3	168	1.79 [0.37; 5.13]
Gutierrez et al. (2004)	1	762	0.13 [0.00; 0.73]
Zehender et al. (2004)	6	167	3.59 [1.33; 7.66]
Zehender et al. (2004)	2	226	0.88 [0.11; 3.16]
Bautista et al. (2009)	3	169	1.78 [0.37; 5.10]
Total (95% CI)		5140	1.17 [0.38; 3.49]
Heterogeneity: $Tau^2 = 1.7437$; $Chi^2 = 100.48$, df = 8 (P < 0.01); $I^2 = 92\%$			
Decade of study = 2002-2011			
Toro et al. (2006)	9	1303	0.69 [0.32; 1.31]
Ramos et al. (2011)	1	1439	0.07 [0.00; 0.39]
Treviño et al. (2011)	6	3337	0.18 [0.07; 0.39]
Total (95% CI)		6079	0.23 [0.02; 2.23]
Heterogeneity: $Tau^2 = 0.5444$; $Chi^2 = 9.45$, df = 2 (P < 0.01); $I^2 = 79\%$			
Decade of study = 2012-2021			
Ramos et al. (2015)	0	157	0.00 [0.00; 2.32]
Bandeira et al. (2015)	2	24	8.33 [1.03; 27.00]
Alessio et al. (2018)	1	1498	0.07 [0.00; 0.37]
Bandeira et al. (2021)	35	320	10.94 [7.74; 14.88]
Abreu et al. (2022)	1	101	0.99 [0.03; 5.39]
Total (95% CI)		2100	0.81 [0.03; 16.37]
Heterogeneity: $Tau^2 = 5.0781$; $Chi^2 = 31.62$, df = 4 (P < 0.01); $I^2 = 87\%$			
**Total (95% CI)**		**15250**	**1.28 [0.58; 2.81]**
Heterogeneity: $Tau^2 = 2.9824$; $Chi^2 = 444.90$, df = 22 (P < 0.01); $I^2 = 95\%$			
Test for subgroup differences: $Chi^2 = 18.48$, df = 3 (P < 0.01)			

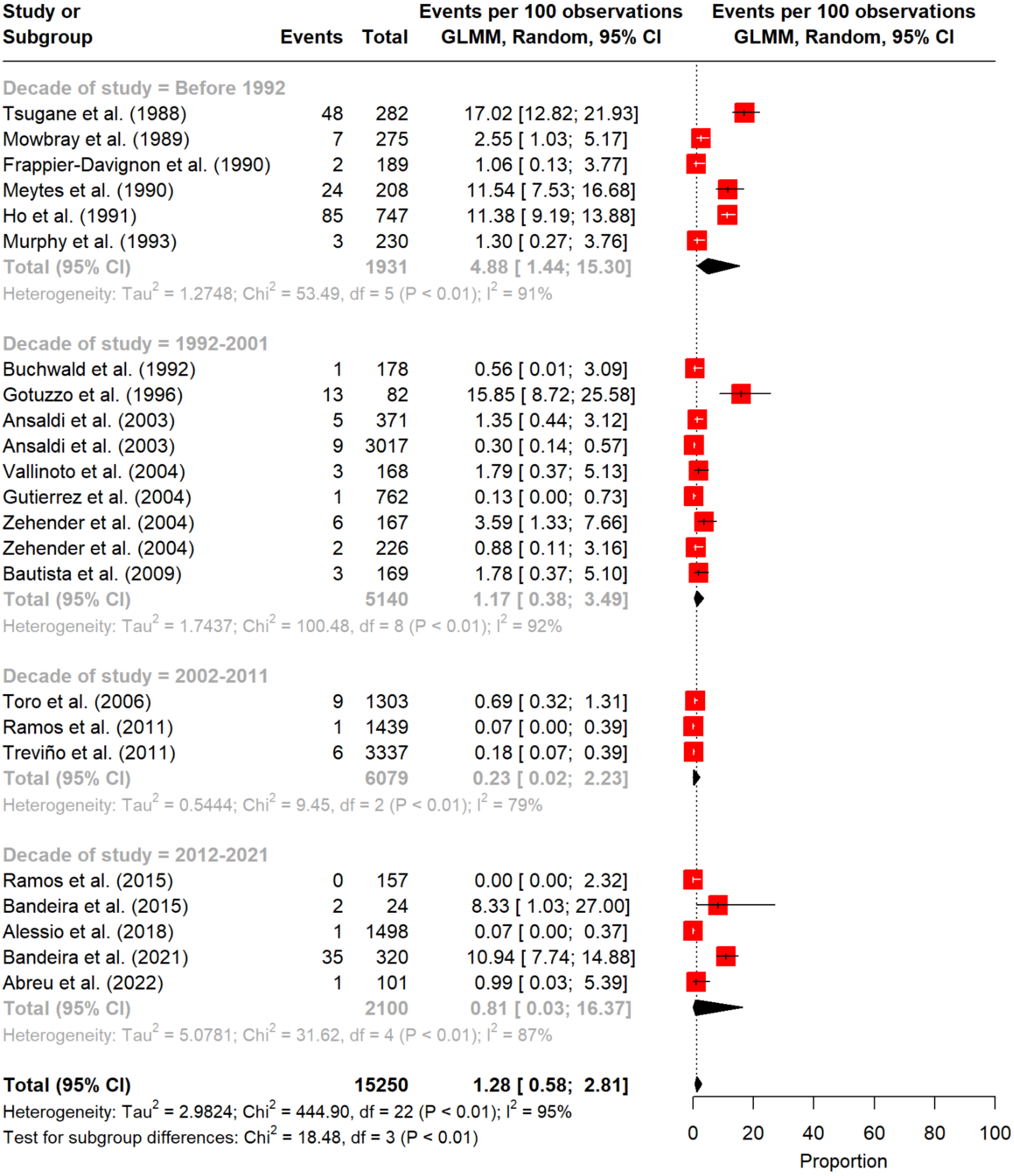

Figure S2. Forest plot of HTLV-1 prevalence in immigrants and refugees by sample size**.**

Study or Subgroup	Events	Total	Events per 100 observations GLMM, Random, 95% CI
**Sample size (n) = <640**			
Tsugane et al. (1988)	48	282	17.02 [12.82; 21.93]
Mowbray et al. (1989)	7	275	2.55 [1.03; 5.17]
Frappier-Davignon et al. (1990)	2	189	1.06 [0.13; 3.77]
Meytes et al. (1990)	24	208	11.54 [7.53; 16.68]
Buchwald et al. (1992)	1	178	0.56 [0.01; 3.09]
Murphy et al. (1993)	3	230	1.30 [0.27; 3.76]
Gotuzzo et al. (1996)	13	82	15.85 [8.72; 25.58]
Ansaldi et al. (2003)	5	371	1.35 [0.44; 3.12]
Vallinoto et al. (2004)	3	168	1.79 [0.37; 5.13]
Zehender et al. (2004)	6	167	3.59 [1.33; 7.66]
Zehender et al. (2004)	2	226	0.88 [0.11; 3.16]
Bautista et al. (2009)	3	169	1.78 [0.37; 5.10]
Ramos et al. (2015)	0	157	0.00 [0.00; 2.32]
Bandeira et al. (2015)	2	24	8.33 [1.03; 27.00]
Bandeira et al. (2021)	35	320	10.94 [7.74; 14.88]
Abreu et al. (2022)	1	101	0.99 [0.03; 5.39]
**Total (95% CI)**		**3147**	**2.62 [1.27; 5.31]**
Heterogeneity: $Tau^2 = 1.5096$; $Chi^2 = 129.06$, df = 15 ($P < 0.01$); $I^2 = 88\%$			
**Sample size (n) = >=640**			
Ho et al. (1991)	85	747	11.38 [9.19; 13.88]
Ansaldi et al. (2003)	9	3017	0.30 [0.14; 0.57]
Gutierrez et al. (2004)	1	762	0.13 [0.00; 0.73]
Toro et al. (2006)	9	1303	0.69 [0.32; 1.31]
Ramos et al. (2011)	1	1439	0.07 [0.00; 0.39]
Treviño et al. (2011)	6	3337	0.18 [0.07; 0.39]
Alessio et al. (2018)	1	1498	0.07 [0.00; 0.37]
**Total (95% CI)**		**12103**	**0.31 [0.05; 1.76]**
Heterogeneity: $Tau^2 = 3.1428$; $Chi^2 = 289.04$, df = 6 ($P < 0.01$); $I^2 = 98\%$			
**Total (95% CI)**		**15250**	**1.28 [0.58; 2.81]**
Heterogeneity: $Tau^2 = 2.9824$; $Chi^2 = 444.90$, df = 22 ($P < 0.01$); $I^2 = 95\%$			
Test for subgroup differences: $Chi^2 = 7.40$, df = 1 ($P < 0.01$)			

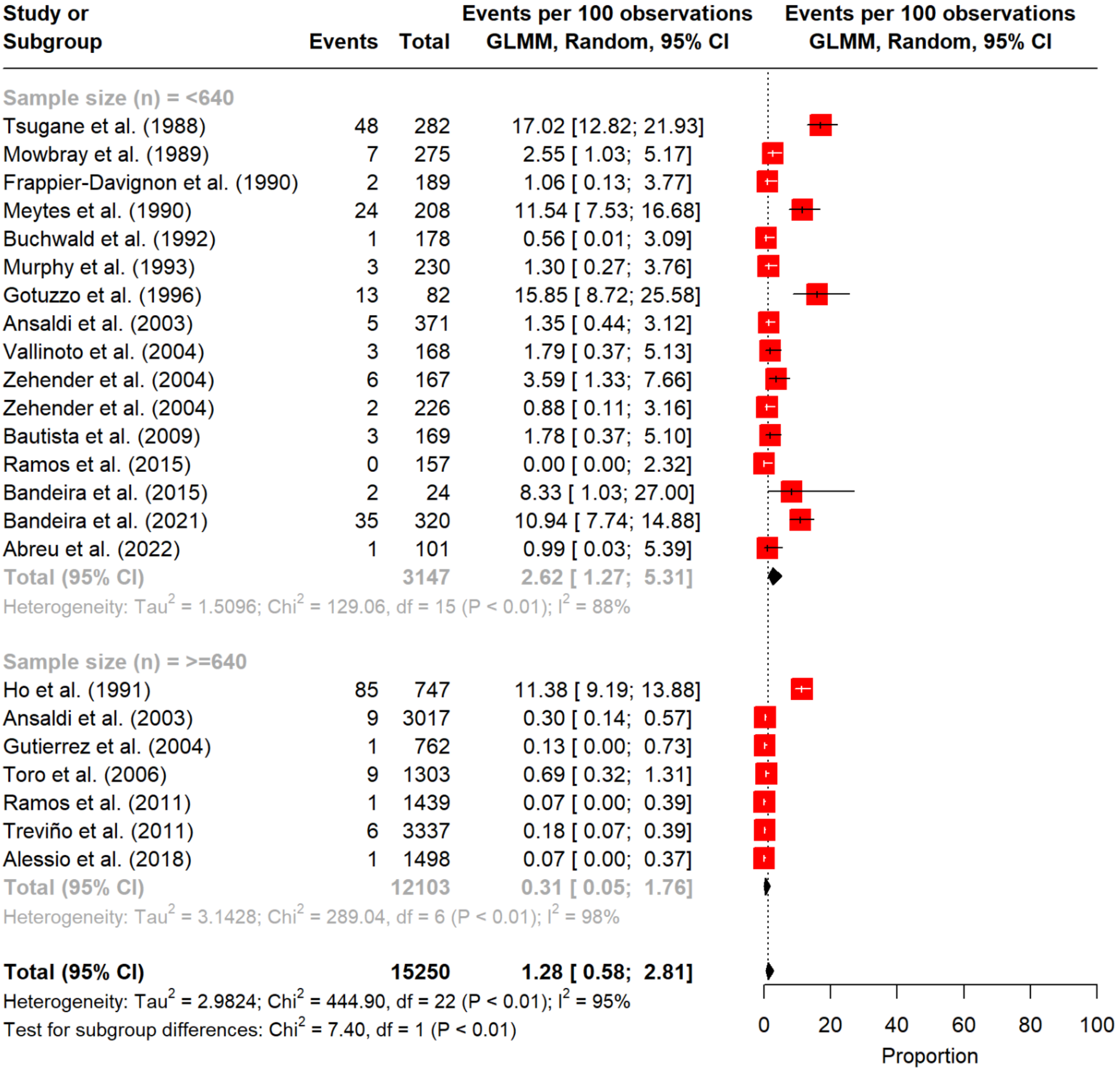

Figure S3. Forest plot of HTLV-1 prevalence in immigrants and refugees by confirmatory methods used.

Study or Subgroup	Events	Total	Events per 100 observations GLMM, Random, 95% CI
**Confirmatory method = PCR or q-PCR**			
Abreu et al. (2022)	1	101	0.99 [0.03; 5.39]
Vallinoto et al. (2004)	3	168	1.79 [0.37; 5.13]
**Total (95% CI)**		**269**	**1.49 [0.00; 90.09]**
Heterogeneity: $Tau^2 = 0$; $Chi^2 = 0.26$, df = 1 (P = 0.61); $I^2 = 0\%$			
**Confirmatory method = WB/LIA & PCR**			
Alessio et al. (2018)	1	1498	0.07 [0.00; 0.37]
Ansaldi et al. (2003)	5	371	1.35 [0.44; 3.12]
Ansaldi et al. (2003)	9	3017	0.30 [0.14; 0.57]
Bandeira et al. (2015)	2	24	8.33 [1.03; 27.00]
Bandeira et al. (2021)	35	320	10.94 [7.74; 14.88]
Meytes et al. (1990)	24	208	11.54 [7.53; 16.68]
Treviño et al. (2011)	6	3337	0.18 [0.07; 0.39]
Zehender et al. (2004)	6	167	3.59 [1.33; 7.66]
Zehender et al. (2004)	2	226	0.88 [0.11; 3.16]
**Total (95% CI)**		**9168**	**1.32 [0.32; 5.25]**
Heterogeneity: $Tau^2 = 3.1291$; $Chi^2 = 214.35$, df = 8 (P < 0.01); $I^2 = 96\%$			
**Confirmatory method = WB**			
Bautista et al. (2009)	3	169	1.78 [0.37; 5.10]
Buchwald et al. (1992)	1	178	0.56 [0.01; 3.09]
Gotuzzo et al. (1996)	13	82	15.85 [8.72; 25.58]
Gutierrez et al. (2004)	1	762	0.13 [0.00; 0.73]
Ho et al. (1991)	85	747	11.38 [9.19; 13.88]
Murphy et al. (1993)	3	230	1.30 [0.27; 3.76]
Ramos et al. (2011)	1	1439	0.07 [0.00; 0.39]
Ramos et al. (2015)	0	157	0.00 [0.00; 2.32]
Toro et al. (2006)	9	1303	0.69 [0.32; 1.31]
Tsugane et al. (1988)	48	282	17.02 [12.82; 21.93]
**Total (95% CI)**		**5349**	**1.15 [0.24; 5.39]**
Heterogeneity: $Tau^2 = 4.2353$; $Chi^2 = 165.16$, df = 9 (P < 0.01); $I^2 = 95\%$			
**Confirmatory method = IFA**			
Frappier-Davignon et al. (1990)	2	189	1.06 [0.13; 3.77]
Mowbray et al. (1989)	7	275	2.55 [1.03; 5.17]
**Total (95% CI)**		**464**	**1.94 [0.03; 58.76]**
Heterogeneity: $Tau^2 = 0$; $Chi^2 = 1.22$, df = 1 (P = 0.27); $I^2 = 18\%$			
**Total (95% CI)**		**15250**	**1.28 [0.58; 2.81]**
Heterogeneity: $Tau^2 = 2.9824$; $Chi^2 = 444.90$, df = 22 (P < 0.01); $I^2 = 95\%$			
Test for subgroup differences: $Chi^2 = 0.69$, df = 3 (P = 0.88)			

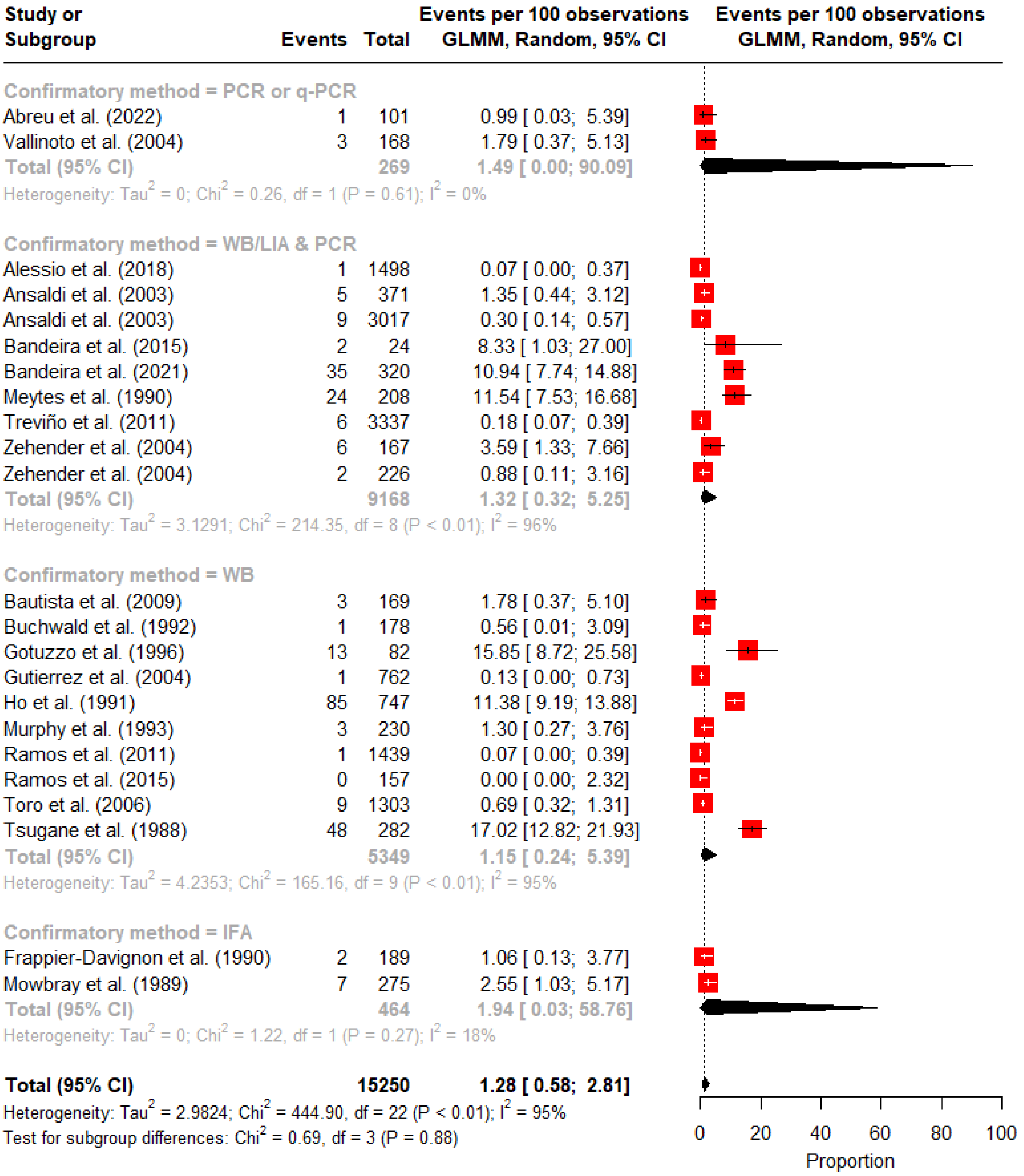

Figure S4. Forest plot of HTLV-1 prevalence in immigrants and refugees by region of study.

Study or Subgroup	Events	Total	Events per 100 observations GLMM, Random, 95% CI
**Continent of the study = Region of the Americas**			
Tsugane et al. (1988)	48	282	17.02 [12.82; 21.93]
Frappier-Davignon et al. (1990)	2	189	1.06 [0.13; 3.77]
Ho et al. (1991)	85	747	11.38 [9.19; 13.88]
Buchwald et al. (1992)	1	178	0.56 [0.01; 3.09]
Gotuzzo et al. (1996)	13	82	15.85 [8.72; 25.58]
Vallinoto et al. (2004)	3	168	1.79 [0.37; 5.13]
Bautista et al. (2009)	3	169	1.78 [0.37; 5.10]
Bandeira et al. (2021)	35	320	10.94 [7.74; 14.88]
Abreu et al. (2022)	1	101	0.99 [0.03; 5.39]
**Total (95% CI)**		**2236**	**3.83 [1.32; 10.60]**
Heterogeneity: Tau^2 = 1.6904; Chi^2 = 61.37, df = 8 (P < 0.01); I^2 = 87%			
**Continent of the study = European Region**			
Mowbray et al. (1989)	7	275	2.55 [1.03; 5.17]
Murphy et al. (1993)	3	230	1.30 [0.27; 3.76]
Ansaldi et al. (2003)	5	371	1.35 [0.44; 3.12]
Ansaldi et al. (2003)	9	3017	0.30 [0.14; 0.57]
Gutierrez et al. (2004)	1	762	0.13 [0.00; 0.73]
Zehender et al. (2004)	6	167	3.59 [1.33; 7.66]
Zehender et al. (2004)	2	226	0.88 [0.11; 3.16]
Toro et al. (2006)	9	1303	0.69 [0.32; 1.31]
Ramos et al. (2011)	1	1439	0.07 [0.00; 0.39]
Treviño et al. (2011)	6	3337	0.18 [0.07; 0.39]
Ramos et al. (2015)	0	157	0.00 [0.00; 2.32]
Bandeira et al. (2015)	2	24	8.33 [1.03; 27.00]
Alessio et al. (2018)	1	1498	0.07 [0.00; 0.37]
**Total (95% CI)**		**12806**	**0.52 [0.21; 1.27]**
Heterogeneity: Tau^2 = 1.7308; Chi^2 = 73.81, df = 12 (P < 0.01); I^2 = 84%			
**Continent of the study = Eastern Mediterranean Region**			
Meytes et al. (1990)	24	208	11.54 [7.53; 16.68]
**Total (95% CI)**		**15250**	**1.28 [0.58; 2.81]**
Heterogeneity: Tau^2 = 2.9824; Chi^2 = 444.90, df = 22 (P < 0.01); I^2 = 95%			
Test for subgroup differences: Chi^2 = 48.35, df = 2 (P < 0.01)			

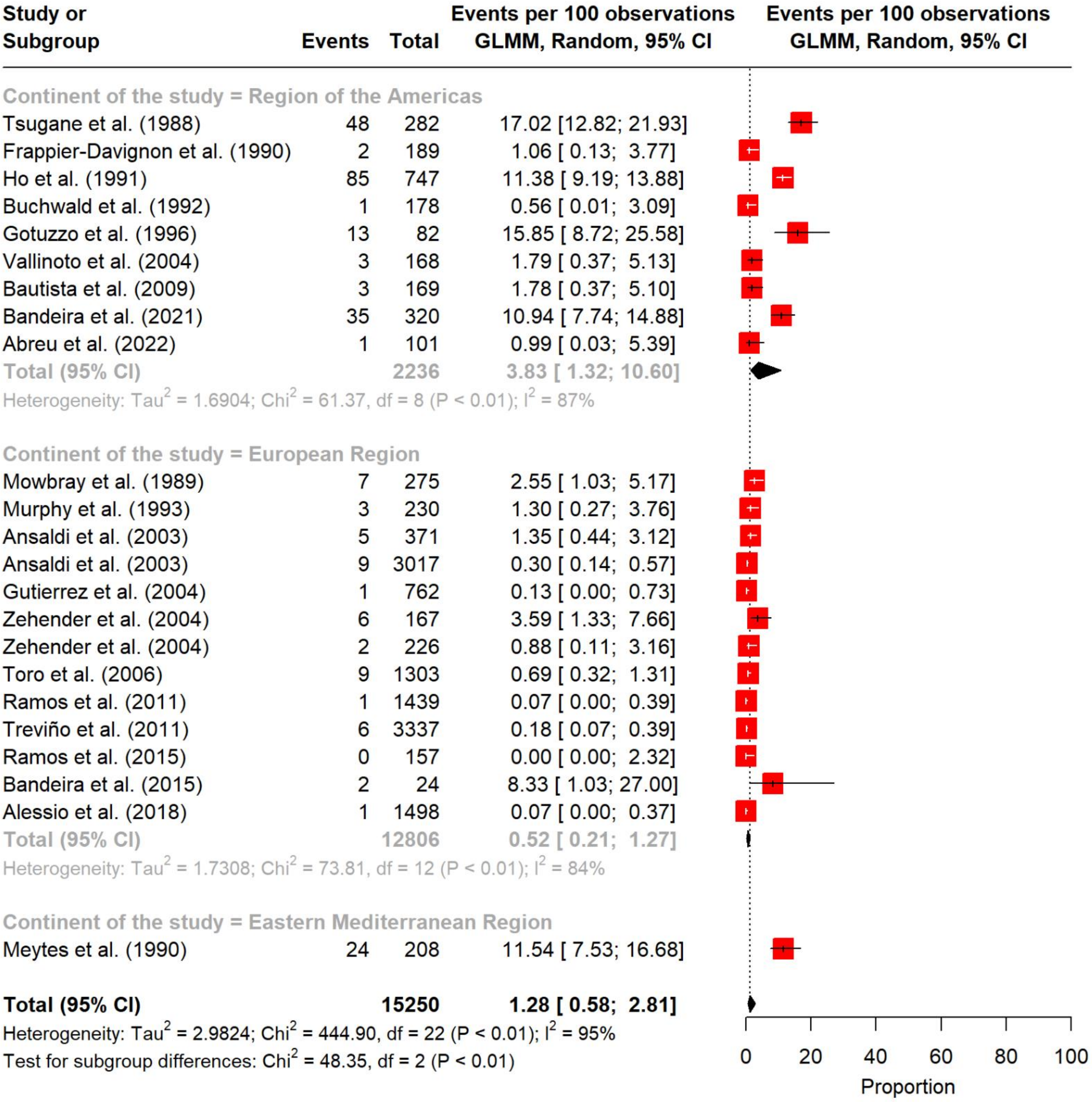

Figure S5. Forest plot of HTLV-1 prevalence in immigrants and refugees by low and high-risk groups.

Study or Subgroup	Events	Total	Events per 100 observations GLMM, Random, 95% CI
**Group = Low-risk**			
Tsugane et al. (1988)	48	282	17.02 [12.82; 21.93]
Mowbray et al. (1989)	7	275	2.55 [1.03; 5.17]
Frappier-Davignon et al. (1990)	2	189	1.06 [0.13; 3.77]
Meytes et al. (1990)	24	208	11.54 [7.53; 16.68]
Ho et al. (1991)	85	747	11.38 [9.19; 13.88]
Buchwald et al. (1992)	1	178	0.56 [0.01; 3.09]
Murphy et al. (1993)	3	230	1.30 [0.27; 3.76]
Gotuzzo et al. (1996)	13	82	15.85 [8.72; 25.58]
Ansaldi et al. (2003)	9	3017	0.30 [0.14; 0.57]
Vallinoto et al. (2004)	3	168	1.79 [0.37; 5.13]
Zehender et al. (2004)	2	226	0.88 [0.11; 3.16]
Toro et al. (2006)	9	1303	0.69 [0.32; 1.31]
Ramos et al. (2011)	1	1439	0.07 [0.00; 0.39]
Treviño et al. (2011)	6	3337	0.18 [0.07; 0.39]
Ramos et al. (2015)	0	157	0.00 [0.00; 2.32]
Bandeira et al. (2015)	2	24	8.33 [1.03; 27.00]
Alessio et al. (2018)	1	1498	0.07 [0.00; 0.37]
Bandeira et al. (2021)	35	320	10.94 [7.74; 14.88]
**Total (95% CI)**		**13680**	**1.37 [0.51; 3.59]**
Heterogeneity: $Tau^2 = 3.5066$; $Chi^2 = 401.84$, df = 17 ($P < 0.01$); $I^2 = 96\%$			
**Group = High-risk**			
Ansaldi et al. (2003)	5	371	1.35 [0.44; 3.12]
Gutierrez et al. (2004)	1	762	0.13 [0.00; 0.73]
Zehender et al. (2004)	6	167	3.59 [1.33; 7.66]
Bautista et al. (2009)	3	169	1.78 [0.37; 5.10]
Abreu et al. (2022)	1	101	0.99 [0.03; 5.39]
**Total (95% CI)**		**1570**	**1.05 [0.27; 3.99]**
Heterogeneity: $Tau^2 = 0.7813$; $Chi^2 = 10.61$, df = 4 ($P = 0.03$); $I^2 = 62\%$			
**Total (95% CI)**		**15250**	**1.28 [0.58; 2.81]**
Heterogeneity: $Tau^2 = 2.9824$; $Chi^2 = 444.90$, df = 22 ($P < 0.01$); $I^2 = 95\%$			
Test for subgroup differences: $Chi^2 = 0.16$, df = 1 ($P = 0.69$)			

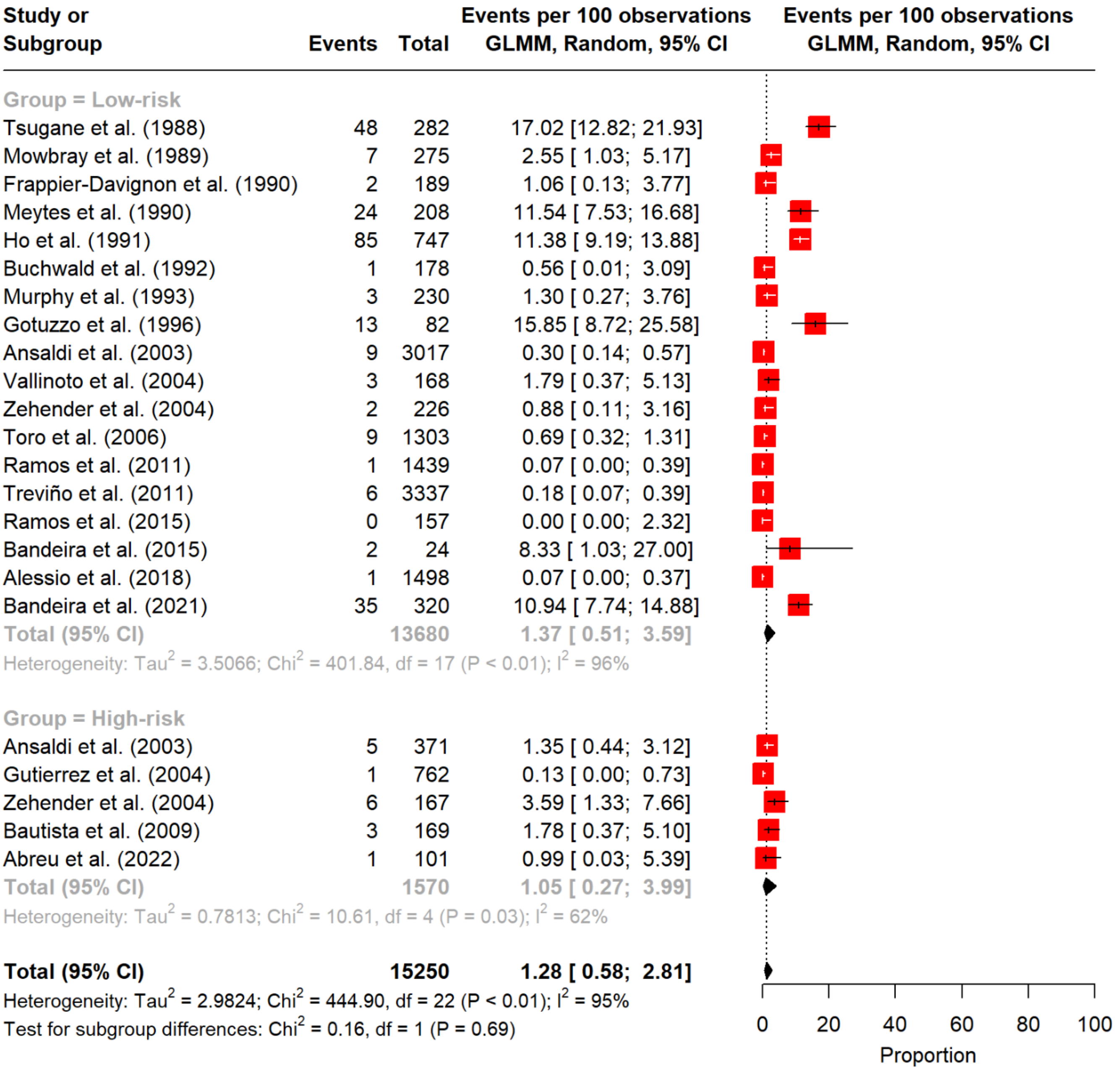

Figure S6. Forest plot of HTLV-2 prevalence in immigrants and refugees by decade of study.

Study or Subgroup	Events	Total	Events per 100 observations GLMM, Random, 95% CI
Decade of study = Before 1992			
Frappier-Davignon et al. (1990)	1	189	0.53 [0.01; 2.91]
Decade of study = 1992-2001			
Ansaldi et al. (2003)	3	371	0.81 [0.17; 2.34]
Ansaldi et al. (2003)	3	3017	0.10 [0.02; 0.29]
Gutierrez et al. (2004)	0	762	0.00 [0.00; 0.48]
Zehender et al. (2004)	2	167	1.20 [0.15; 4.26]
Zehender et al. (2004)	0	226	0.00 [0.00; 1.62]
Bautista et al. (2009)	0	169	0.00 [0.00; 2.16]
Total (95% CI)		4712	0.16 [0.03; 1.04]
Heterogeneity: Tau^2 = 1.3058; Chi^2 = 9.73, df = 5 (P = 0.08); I^2 = 49%			
Decade of study = 2002-2011			
Toro et al. (2006)	1	1303	0.08 [0.00; 0.43]
Ramos et al. (2011)	2	1439	0.14 [0.02; 0.50]
Treviño et al. (2011)	1	3337	0.03 [0.00; 0.17]
Total (95% CI)		6079	0.07 [0.01; 0.56]
Heterogeneity: Tau^2 = 0; Chi^2 = 1.58, df = 2 (P = 0.45); I^2 = 0%			
Decade of study = 2012-2021			
Ramos et al. (2015)	0	157	0.00 [0.00; 2.32]
Bandeira et al. (2015)	0	24	0.00 [0.00; 14.25]
Alessio et al. (2018)	0	1498	0.00 [0.00; 0.25]
Bandeira et al. (2021)	0	320	0.00 [0.00; 1.15]
Abreu et al. (2022)	2	101	1.98 [0.24; 6.97]
Total (95% CI)		2100	0.01 [0.00; 62.93]
Heterogeneity: Tau^2 = 10.4183; Chi^2 = 0, df = 4 (P = 1.00); I^2 = 0%			
**Total (95% CI)**		**13080**	**0.11 [0.04; 0.33]**
Heterogeneity: Tau^2 = 1.7233; Chi^2 = 26.11, df = 14 (P = 0.03); I^2 = 46%			
Test for subgroup differences: Chi^2 = 4.20, df = 3 (P = 0.24)			

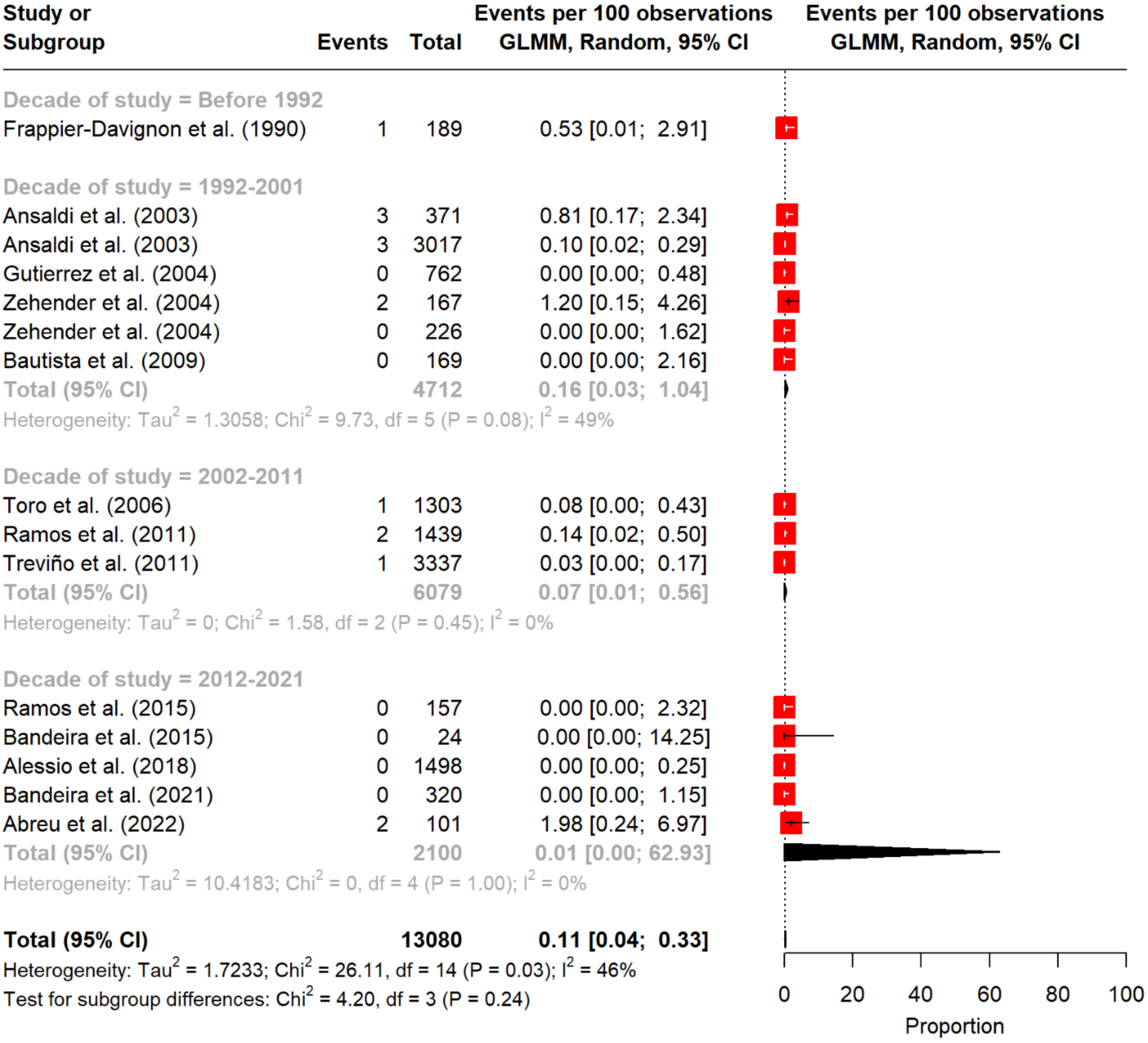

Figure S7. Forest plot of HTLV-2 prevalence in immigrants and refugees by sample size.

Study or Subgroup	Events	Total	Events per 100 observations GLMM, Random, 95% CI
**Sample size (n) = <640**			
Frappier-Davignon et al. (1990)	1	189	0.53 [0.01; 2.91]
Ansaldi et al. (2003)	3	371	0.81 [0.17; 2.34]
Zehender et al. (2004)	2	167	1.20 [0.15; 4.26]
Zehender et al. (2004)	0	226	0.00 [0.00; 1.62]
Bautista et al. (2009)	0	169	0.00 [0.00; 2.16]
Ramos et al. (2015)	0	157	0.00 [0.00; 2.32]
Bandeira et al. (2015)	0	24	0.00 [0.00; 14.25]
Bandeira et al. (2021)	0	320	0.00 [0.00; 1.15]
Abreu et al. (2022)	2	101	1.98 [0.24; 6.97]
**Total (95% CI)**		**1724**	**0.40 [0.11; 1.39]**
Heterogeneity: $Tau^2 = 0.3582$; $Chi^2 = 1.53$, df = 8 (P = 0.99); $I^2 = 0\%$			
**Sample size (n) = >=640**			
Ansaldi et al. (2003)	3	3017	0.10 [0.02; 0.29]
Gutierrez et al. (2004)	0	762	0.00 [0.00; 0.48]
Toro et al. (2006)	1	1303	0.08 [0.00; 0.43]
Ramos et al. (2011)	2	1439	0.14 [0.02; 0.50]
Treviño et al. (2011)	1	3337	0.03 [0.00; 0.17]
Alessio et al. (2018)	0	1498	0.00 [0.00; 0.25]
**Total (95% CI)**		**11356**	**0.06 [0.02; 0.16]**
Heterogeneity: $Tau^2 = 0$; $Chi^2 = 1.64$, df = 5 (P = 0.90); $I^2 = 0\%$			
**Total (95% CI)**		**13080**	**0.11 [0.04; 0.33]**
Heterogeneity: $Tau^2 = 1.7233$; $Chi^2 = 26.11$, df = 14 (P = 0.03); $I^2 = 46\%$			
Test for subgroup differences: $Chi^2 = 7.99$, df = 1 (P < 0.01)			

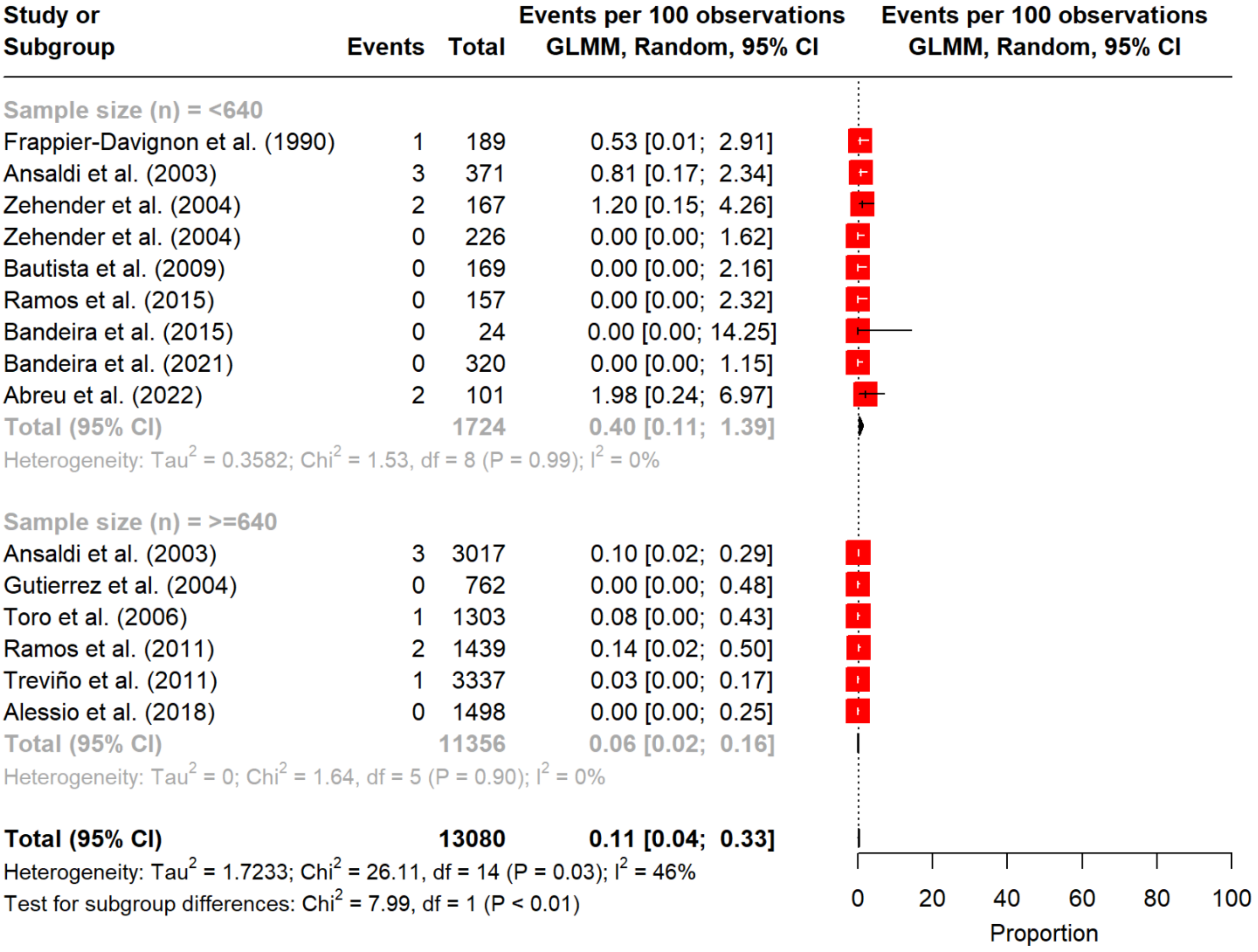

Figure S8. Forest plot of HTLV-2 prevalence in immigrants and refugees by confirmatory methods used.

Study or Subgroup	Events	Total	Events per 100 observations GLMM, Random, 95% CI
Confirmatory method = PCR or qPCR			
Abreu et al. (2022)	2	101	1.98 [0.24; 6.97]
Confirmatory method = WB/LIA & PCR			
Alessio et al. (2018)	0	1498	0.00 [0.00; 0.25]
Ansaldi et al. (2003)	3	371	0.81 [0.17; 2.34]
Ansaldi et al. (2003)	3	3017	0.10 [0.02; 0.29]
Bandeira et al. (2015)	0	24	0.00 [0.00; 14.25]
Bandeira et al. (2021)	0	320	0.00 [0.00; 1.15]
Treviño et al. (2011)	1	3337	0.03 [0.00; 0.17]
Zehender et al. (2004)	2	167	1.20 [0.15; 4.26]
Zehender et al. (2004)	0	226	0.00 [0.00; 1.62]
Total (95% CI)		8960	0.09 [0.01; 0.57]
Heterogeneity: Tau^2 = 2.2169; Chi^2 = 15.78, df = 7 (P = 0.03); I^2 = 56%			
Confirmatory method = WB			
Bautista et al. (2009)	0	169	0.00 [0.00; 2.16]
Gutierrez et al. (2004)	0	762	0.00 [0.00; 0.48]
Ramos et al. (2011)	2	1439	0.14 [0.02; 0.50]
Ramos et al. (2015)	0	157	0.00 [0.00; 2.32]
Toro et al. (2006)	1	1303	0.08 [0.00; 0.43]
Total (95% CI)		3830	0.08 [0.02; 0.39]
Heterogeneity: Tau^2 = 0; Chi^2 = 0.24, df = 4 (P = 0.99); I^2 = 0%			
Confirmatory method = IFA			
Frappier-Davignon et al. (1990)	1	189	0.53 [0.01; 2.91]
**Total (95% CI)**		**13080**	**0.11 [0.04; 0.33]**
Heterogeneity: Tau^2 = 1.7233; Chi^2 = 26.11, df = 14 (P = 0.03); I^2 = 46%			
Test for subgroup differences: Chi^2 = 14.90, df = 3 (P < 0.01)			

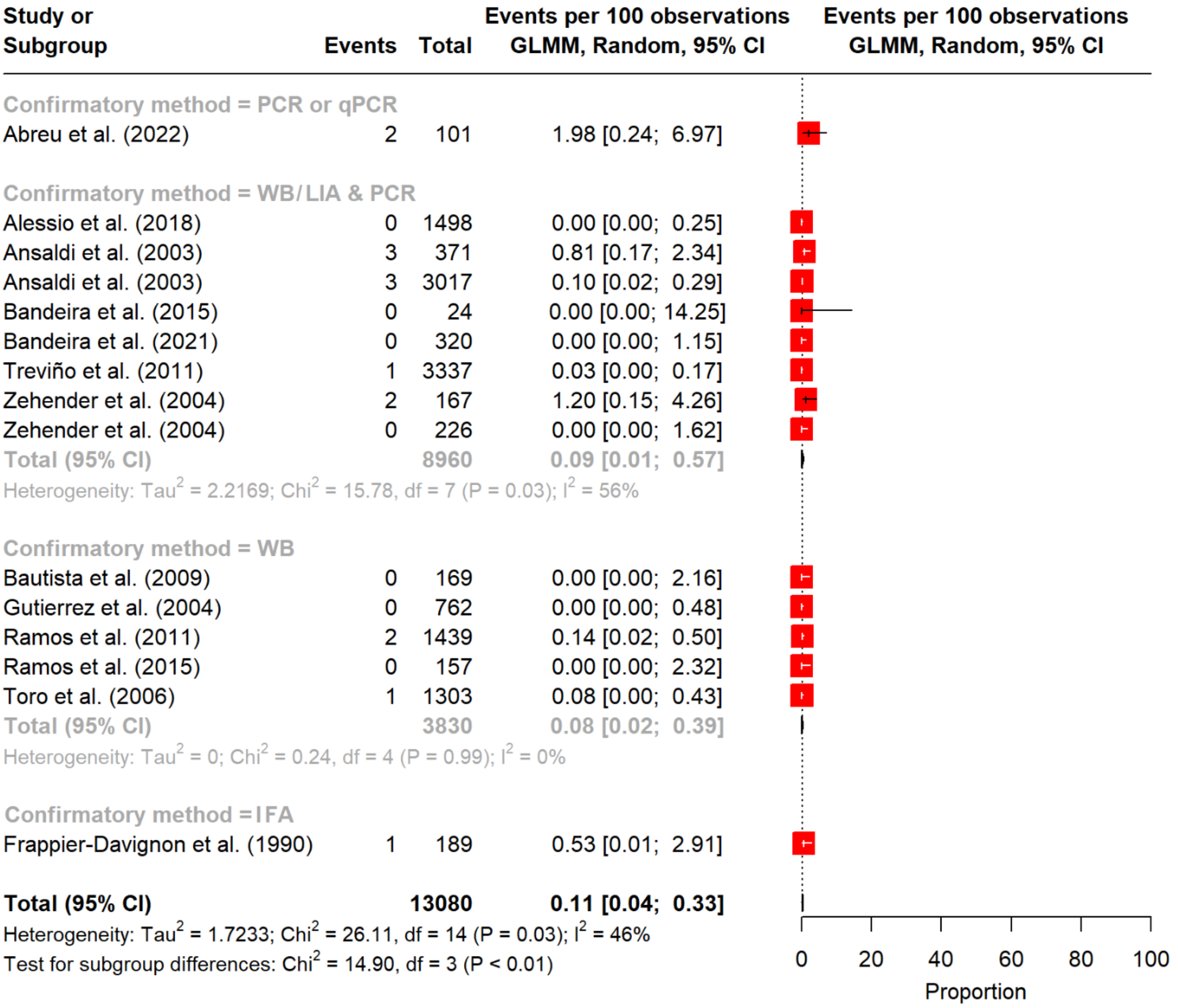

Figure S9. Forest plot of HTLV-2 prevalence in immigrants and refugees by region of study.

Study or Subgroup	Events	Total	Events per 100 observations GLMM, Random, 95% CI
**Continent of the study = Region of the Americas**			
Frappier-Davignon et al. (1990)	1	189	0.53 [0.01; 2.91]
Bautista et al. (2009)	0	169	0.00 [0.00; 2.16]
Bandeira et al. (2021)	0	320	0.00 [0.00; 1.15]
Abreu et al. (2022)	2	101	1.98 [0.24; 6.97]
**Total (95% CI)**		**779**	**0.29 [0.01; 6.39]**
Heterogeneity: $Tau^2 = 1.1174$; $Chi^2 = 1.18$, df = 3 (P = 0.76); $I^2 = 0\%$			
**Continent of the study = European Region**			
Ansaldi et al. (2003)	3	371	0.81 [0.17; 2.34]
Ansaldi et al. (2003)	3	3017	0.10 [0.02; 0.29]
Gutierrez et al. (2004)	0	762	0.00 [0.00; 0.48]
Zehender et al. (2004)	2	167	1.20 [0.15; 4.26]
Zehender et al. (2004)	0	226	0.00 [0.00; 1.62]
Toro et al. (2006)	1	1303	0.08 [0.00; 0.43]
Ramos et al. (2011)	2	1439	0.14 [0.02; 0.50]
Treviño et al. (2011)	1	3337	0.03 [0.00; 0.17]
Ramos et al. (2015)	0	157	0.00 [0.00; 2.32]
Bandeira et al. (2015)	0	24	0.00 [0.00; 14.25]
Alessio et al. (2018)	0	1498	0.00 [0.00; 0.25]
**Total (95% CI)**		**12301**	**0.09 [0.03; 0.30]**
Heterogeneity: $Tau^2 = 1.3509$; $Chi^2 = 18.18$, df = 10 (P = 0.05); $I^2 = 45\%$			
**Total (95% CI)**		**13080**	**0.11 [0.04; 0.33]**
Heterogeneity: $Tau^2 = 1.7233$; $Chi^2 = 26.11$, df = 14 (P = 0.03); $I^2 = 46\%$			
Test for subgroup differences: $Chi^2 = 1.17$, df = 1 (P = 0.28)			

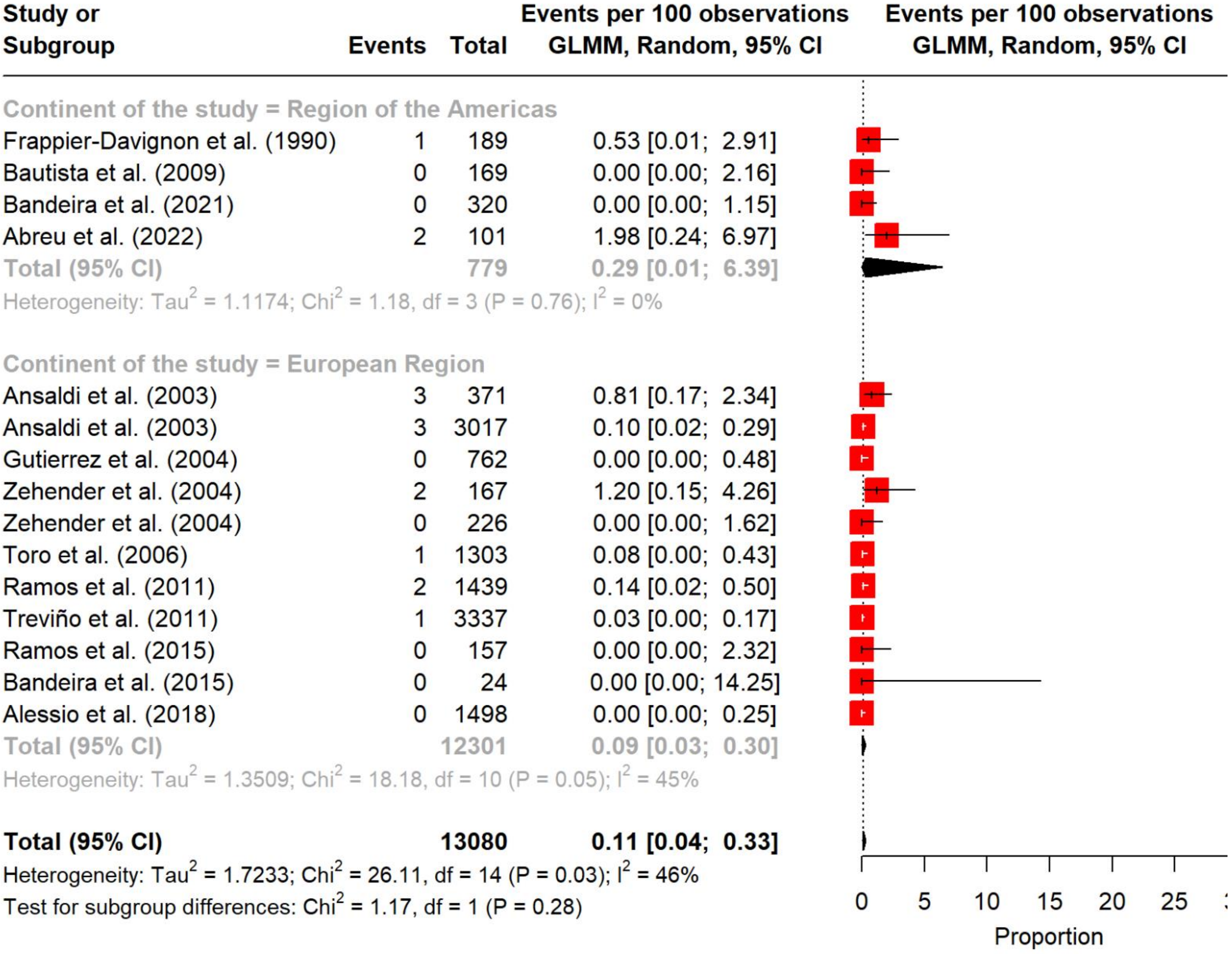

Figure S10. Forest plot of HTLV-2 prevalence in immigrants and refugees by low and high-risk groups.

Study or Subgroup	Events	Total	Events per 100 observations GLMM, Random, 95% CI
**Group = Low-risk**			
Frappier-Davignon et al. (1990)	1	189	0.53 [0.01; 2.91]
Ansaldi et al. (2003)	3	3017	0.10 [0.02; 0.29]
Zehender et al. (2004)	0	226	0.00 [0.00; 1.62]
Toro et al. (2006)	1	1303	0.08 [0.00; 0.43]
Ramos et al. (2011)	2	1439	0.14 [0.02; 0.50]
Treviño et al. (2011)	1	3337	0.03 [0.00; 0.17]
Ramos et al. (2015)	0	157	0.00 [0.00; 2.32]
Bandeira et al. (2015)	0	24	0.00 [0.00; 14.25]
Alessio et al. (2018)	0	1498	0.00 [0.00; 0.25]
Bandeira et al. (2021)	0	320	0.00 [0.00; 1.15]
**Total (95% CI)**		**11510**	**0.07 [0.03; 0.15]**
Heterogeneity: $Tau^2 = 0$; $Chi^2 = 4.43$, df = 9 (P = 0.88); $I^2 = 0\%$			
**Group = High-risk**			
Ansaldi et al. (2003)	3	371	0.81 [0.17; 2.34]
Gutierrez et al. (2004)	0	762	0.00 [0.00; 0.48]
Zehender et al. (2004)	2	167	1.20 [0.15; 4.26]
Bautista et al. (2009)	0	169	0.00 [0.00; 2.16]
Abreu et al. (2022)	2	101	1.98 [0.24; 6.97]
**Total (95% CI)**		**1570**	**0.40 [0.05; 2.89]**
Heterogeneity: $Tau^2 = 1.2640$; $Chi^2 = 0.98$, df = 4 (P = 0.91); $I^2 = 0\%$			
**Total (95% CI)**		**13080**	**0.11 [0.04; 0.33]**
Heterogeneity: $Tau^2 = 1.7233$; $Chi^2 = 26.11$, df = 14 (P = 0.03); $I^2 = 46\%$			
Test for subgroup differences: $Chi^2 = 4.74$, df = 1 (P = 0.03)			

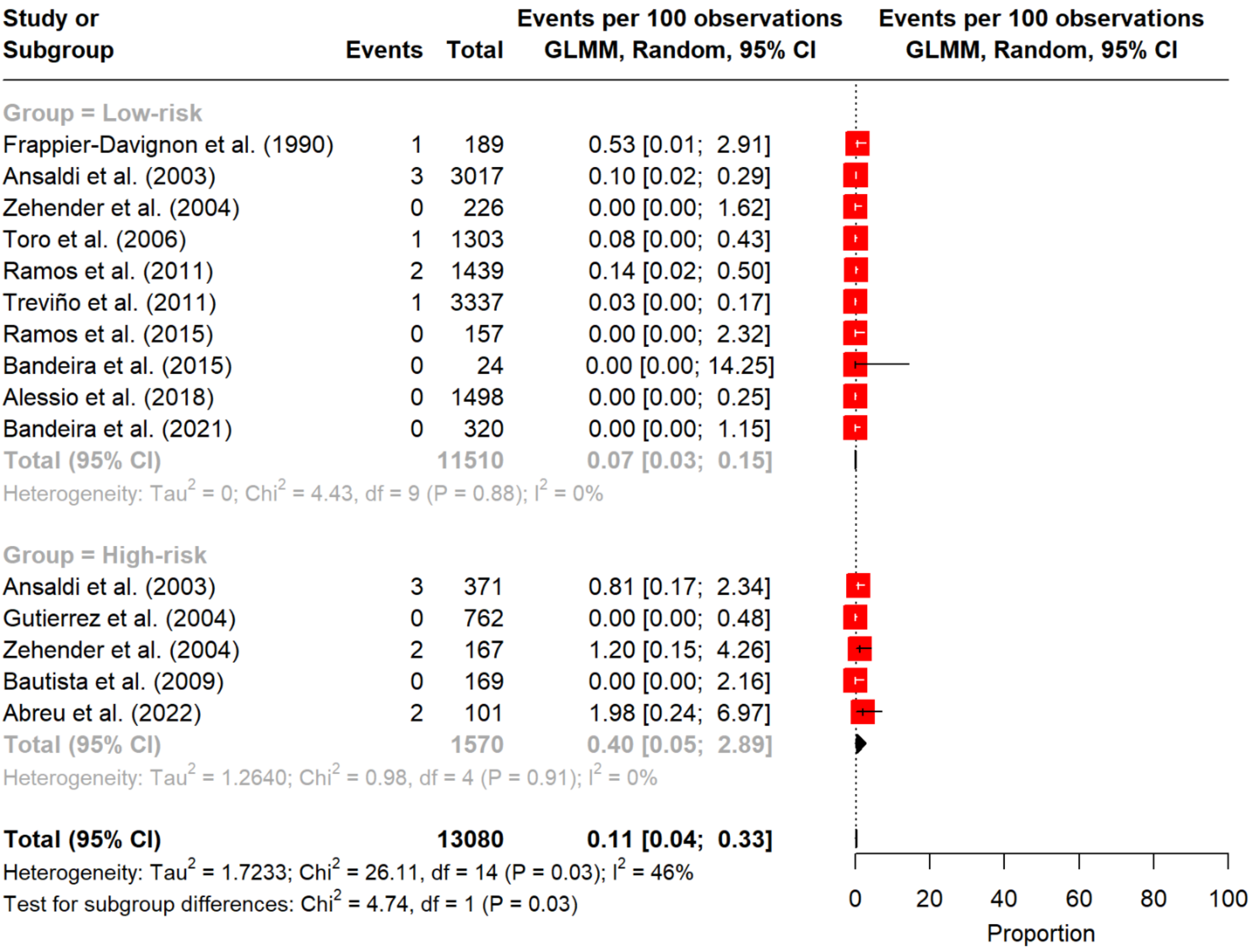

Supplement: Supplementary file 1 [file viruses-16-01526-s001.zip › Figures S1 - S10.pdf]
