# Supplementary material for: The Global Prevalence of HTLV-1 and HTLV-2 Infections among Immigrants and Refugees—A Systematic Review and Meta-Analysis
Source: Viruses. 2024 Sep 27;16(10):1526. doi: 10.3390/v16101526 (PMC11512286; doi:10.3390/v16101526)
Supplement: Supplementary file 1 [file viruses-16-01526-s001.zip › Table S2 Search strategies from the PubMed, Scopus, EMBASE, Web of Science and VHL databases.pdf]

Table S2: Search strategies from the PubMed, Scopus, EMBASE, Web of Science and VHL databases

MEDLINE (via PUBMED)
(((((((((Emigrants and Immigrants[MeSH Terms]) OR (Foreigners[Title/Abstract])) OR (Alien[Title/Abstract])) OR (Immigrant*[Title/Abstract])) OR (Refugees[Title/Abstract])) OR (Asylum Seekers[Title/Abstract])) OR (Seeker Asylum[Title/Abstract])) OR (Person, Displaced[Title/Abstract])) OR (Refugee*[Title/Abstract])) AND ((((Human T-lymphotropic virus 1[MeSH Terms]) OR (HTLV-*[Title/Abstract])) OR (Human T-lymphotropic virus 2[MeSH Terms])) OR (Leukemia Lymphoma Virus 1 Adult T Cell[Title/Abstract]))) AND (((((((Prevalence[MeSH Terms]) OR (Prevalence[Title/Abstract])) OR (Epidemiology[MeSH Terms])) OR (Epidemiology[Title/Abstract])) OR (Epidemiologies Social[Title/Abstract])) OR (Cross-Sectional Studies[MeSH Terms])) OR (Cross-Sectional*[Title/Abstract]))
Scopus
(TITLE-ABS (human-t-lymphotropic-virus-2) OR TITLE-ABS (htlv-*) OR TITLE-ABS (leukemia-lymphoma-virus-1,adult-t-cell) OR TITLE-ABS (human-t-lymphotropic-virus-1)) AND (TITLE-ABS (cross-sectional*) OR TITLE-ABS (cross-sectional-study) OR TITLE-ABS (epidemiologies-social) OR TITLE-ABS (epidemiology) OR TITLE-ABS (prevalence)) AND (TITLE-ABS (refugee*) OR TITLE-ABS (person AND displaced) OR TITLE-ABS (seeker AND asylum) OR (asylum AND seekers) OR TITLE-ABS (immigrant*) OR TITLE-ABS (alien) OR TITLE-ABS (foreigners) OR TITLE-ABS (refugees) OR TITLE-ABS (immigrants) OR TITLE-ABS (emigrants))
EMBASE
immigrants:ti,ab,kw OR foreigner:ti,ab,kw OR refugee:ti,ab,kw OR 'asylum seeker':ti,ab,kw OR 'seeker, asylum':ti,ab,kw OR 'person, displaced':ti,ab,kw AND 'human t-lymphotropic virus 1':ti,ab,kw OR 'leukemia lymphoma virus 1, adult t cell':ti,ab,kw OR htlv:ti,ab,kw OR 'human t-lymphotropic virus 2':ti,ab,kw AND prevalence:ti,ab,kw OR epidemiology:ti,ab,kw OR 'epidemiologies, social':ti,ab,kw OR 'cross-sectional study':ti,ab,kw OR 'cross sectional':ti,ab,kw
Web of Science
TS=(prevalence) OR TS=(epidemiology) OR TI=(epidemiologies social) OR TS=(cross-sectional studies) OR TI=(cross-sectional*) Índices=SCI-EXPANDED, SSCI, A&HCI, CPCI-S, CPCI-SSH, ESCI Tempo estipulado=Todos os anos AND TS=(human t-lymphotropic virus 1) OR TI=(leukemia lymphoma virus 1, adult t cell) OR TI=(htlv-*) OR TS=(human t-lymphotropic virus 2) Índices=SCI-EXPANDED, SSCI, A&HCI, CPCI-S, CPCI-SSH, ESCI Tempo estipulado=Todos os anos AND TS=(immigrant*) OR TS=(refugees) OR TI=(foreigners) OR TI=(alien) OR TI=(asylum seekers) OR TI=(seeker, asylum) OR TI=(person, displaced) OR TI=(refugee*) Índices=SCI-EXPANDED, SSCI, A&HCI, CPCI-S, CPCI-SSH, ESCI Tempo estipulado=Todos os anos
Virtual Health Library (VHL)/Biblioteca Virtual em Saúde (BVS)

((Emigrantes e Imigrantes) OR (Alienígenas) OR (Estrangeiros) OR (Imigrantes) OR ((mh:(Refugiados)) OR (Refugiados) OR (Pessoas Deslocadas) OR (Requerentes de Asilo)) AND (((mh:(Vírus Linfotrópico T Tipo 1 Humano)) OR (Vírus Linfotrópico T Tipo 1 Humano) OR (Vírus Linfotrópico T Tipo 1 Humano) OR (Vírus I da Leucemia-Linfoma das Células T do Adulto) OR (HTLV-*) OR (mh:(Vírus Linfotrópico T Tipo 2 Humano)) OR (Vírus Linfotrópico T Tipo 2 Humano)) AND ((mh:(Prevalência)) OR (Prevalência) OR (mh:(Epidemiologia)) OR (Epidemiologia) OR (Epidemiologia Social) OR (mh:(Estudos Transversais)) OR (Estudos Transversais) OR (Estudo Transversal)))
