# Supplementary material for: The Global Prevalence of HTLV-1 and HTLV-2 Infections among Immigrants and Refugees—A Systematic Review and Meta-Analysis
Source: Viruses. 2024 Sep 27;16(10):1526. doi: 10.3390/v16101526 (PMC11512286; doi:10.3390/v16101526)
Supplement: Supplementary file 1 [file viruses-16-01526-s001.zip › TABLES~S3.pdf]

TABLE S3. Assessment of quality of HTLV-1/2 prevalence studies in international migrants included in this systematic review according to the JBI Critical Appraisal Checklist for Analytical Cross Sectional Studies.

Reference	Q1	Q2	Q3	Q4	Q5	Q6	Q7	Q8	Total
**European Region**									
Alessio et al., 2018	Y	Y	Y	Y	N/A	N/A	Y	N/A	5
Ramos et al., 2015	Y	Y	Y	Y	N/A	N/A	Y	N/A	5
Ramos et al., 2011	Y	Y	Y	Y	N/A	N/A	Y	N/A	5
Treviño et al., 2011	Y	Y	Y	Y	N/A	N/A	Y	N/A	5
Toro et al., 2006	Y	Y	Y	Y	N/A	N/A	Y	N/A	5
Gutierrez et al., 2004	Y	Y	Y	Y	N/A	N/A	Y	N/A	5
Ansaldi et al., 2003	Y	Y	Y	Y	N/A	N/A	Y	N/A	5
Mowbray et al., 1989	Y	Y	Y	Y	N/A	N/A	Y	N/A	5
**Eastern Mediterranean Region**									
Meytes et al., 1990	Y	Y	Y	Y	N/A	N/A	Y	N/A	5
**Region of the Americas (North America)**									
Murphy et al., 1993	Y	Y	Y	Y	N/A	N/A	Y	N/A	5
Buchwald et al., 1992	Y	Y	Y	Y	N/A	N/A	Y	N/A	5
Ho et al., 1991	Y	Y	Y	Y	N/A	N/A	Y	N/A	5
Frappier-Davignon et al.,1990	N	Y	N	Y	Y	N	N	Y	Y
**Region of the Americas (South America)**									
Abreu et al. 2022	Y	Y	Y	Y	N/A	N/A	Y	N/A	5
Bandeira et al., 2021	Y	Y	Y	Y	N/A	N/A	Y	N/A	5
Bandeira et al., 2015	Y	Y	Y	Y	N/A	N/A	Y	N/A	5
Bautista et al. 2009	Y	Y	Y	Y	N/A	N/A	Y	N/A	5
Vallinoto et al., 2004	Y	Y	Y	Y	N/A	N/A	Y	N/A	5
Gotuzzo et al., 1996	Y	Y	Y	Y	N/A	N/A	Y	N/A	5
Tsugane et al., 1988	Y	Y	Y	Y	N/A	N/A	Y	N/A	5

N, no; N/A, not applicable; U, unclear; Y, yes.
Q1: Were the criteria for inclusion in the sample clearly defined?
Q2: Were the study subjects and the setting described in detail?
Q3: Was the exposure measured in a valid and reliable way?
Q4: Were objective, standard criteria used for measurement of the condition?
Q5: Were confounding factors identified?
Q6: Were strategies to deal with confounding factors stated?
Q7: Were the outcomes measured in a valid and reliable way?
Q8: Was appropriate statistical analysis used?
