# Supplementary material for: The Global Prevalence of HTLV-1 and HTLV-2 Infections among Immigrants and Refugees—A Systematic Review and Meta-Analysis
Source: Viruses. 2024 Sep 27;16(10):1526. doi: 10.3390/v16101526 (PMC11512286; doi:10.3390/v16101526)
Supplement: Supplementary file 1 [file viruses-16-01526-s001.zip › TABLES~S4.pdf]

**TABLE S4.** Assessment of quality of studies of HTLV-1/2 prevalence in immigrants included in the systematic review according to the JBI Critical Appraisal Checklist for Case Control Studies.

| Reference | Q1 | Q2 | Q3 | Q4 | Q5 | Q6 | Q7 | Q8 | Q9 | Q10 | Total |
|---|---|---|---|---|---|---|---|---|---|---|---|
| **European Region** | | | | | | | | | | | |
| Zehender et al., 2004 | N | Y | N | Y | Y | N | N | Y | Y | N/A | 5 |

N, no; N/A, not applicable; U, unclear; Y, yes.
Q1: Were the groups comparable other than the presence of disease in cases or the absence of disease in controls?
Q2: Were cases and controls matched appropriately?
Q3: Were the same criteria used for identification of cases and controls?
Q4: Was exposure measured in a standard, valid and reliable way?
Q5: Was exposure measured in the same way for cases and controls?
Q6: Were confounding factors identified?
Q7: Were strategies to deal with confounding factors stated?
Q8: Were outcomes assessed in a standard, valid and reliable way for cases and controls?
Q9: Was the exposure period of interest long enough to be meaningful?
Q10: Was appropriate statistical analysis used?
